# Implicit and explicit influences of religious cognition on Dictator Game transfers

**DOI:** 10.1098/rsos.170238

**Published:** 2018-08-22

**Authors:** Joseph Billingsley, Cristina M. Gomes, Michael E. McCullough

**Affiliations:** Department of Psychology, University of Miami, Coral Gables, FL, USA

**Keywords:** religious priming, Dictator Game, religion, cooperation, prosocial

## Abstract

Does religion promote prosocial behaviour? Despite numerous publications that seem to answer this question affirmatively, divergent results from recent meta-analyses and pre-registered replication efforts suggest that the issue is not yet settled. Uncertainty lingers around (i) whether the effects of religious cognition on prosocial behaviour were obtained through implicit cognitive processes, explicit cognitive processes or both and (ii) whether religious cognition increases generosity only among people disinclined to share with anonymous strangers. Here, we report two experiments designed to address these concerns. In Experiment 1, we sought to replicate Shariff and Norenzayan's demonstration of the effects of implicit religious priming on Dictator Game transfers to anonymous strangers; unlike Shariff and Norenzayan, however, we used an online environment where anonymity was virtually assured. In Experiment 2, we introduced a ‘taking’ option to allow greater expression of baseline selfishness. In both experiments, we sought to activate religious cognition implicitly and explicitly, and we investigated the possibility that religious priming depends on the extent to which subjects view God as a punishing, authoritarian figure. Results indicated that in both experiments, religious subjects transferred more money on average than did non-religious subjects. Bayesian analyses supported the null hypothesis that *implicit* religious priming did not increase Dictator Game transfers in either experiment, even among religious subjects. Collectively, the two experiments furnished support for a small but reliable effect of *explicit* priming, though among religious subjects only. Neither experiment supported the hypothesis that the effect of religious priming depends on viewing God as a punishing figure. Finally, in a meta-analysis of relevant studies, we found that the overall effect of implicit religious priming on Dictator Game transfers was small and did not statistically differ from zero.

## 1. Introduction

Prosocial encouragement features prominently in the world's major religions [1]. These religions urge adherents to love neighbours or even strangers as they would themselves (e.g. Leviticus 19:18; Leviticus 19:34; Mark 12:31), to provide charity to those in need (e.g. Surah Al-Baqarah 2:83; Mark 10:17–23) and to act toward others as they would have others act toward them (e.g. Matthew 7:12; Talmud Shabbat 31a).

This association of world religions with benevolence toward others has motivated, in part, the development of at least two related bodies of theory. First is the ‘religious prosociality hypothesis' [2, p. 876], defined variously as the notion that ‘religions facilitate costly behaviors that benefit other people’ [3, p. 58] or that ‘religious belief or concepts lead to prosocial attitudes and behaviors' [2, p. 876]. Second is a broader set of claims fashioned into a cultural evolutionary model of religious prosociality [4,5] (for related proposals, see also [6–9]). According to this model, cultures have varied in the extent to which deities and other supernatural agents are understood to involve themselves in human affairs and care about prosociality or other moralized behaviours. Past cultures marked by supernatural agents of relatively greater power and heightened interest in moralizing human behaviour—‘Big Gods’ for short [4]—would have experienced higher levels of prosocial behaviour compared to cultures with weak, morally indifferent deities. In turn, the increased levels of prosociality promoted by powerful moralizing deities would have facilitated the emergence of large-scale societies where cooperation occurs even within groups of individuals who are not closely related by recent ancestry and who do not regularly interact [5]. In addition, increased levels of prosociality would have fostered greater social solidarity and ultimately greater success in competition with other cultures [5]. If true, this model would thus help account simultaneously for the rise of large-scale cooperative societies, and for the prevalence of prosocial norms across the contemporary religious landscape [5].

An impressive array of research has been brought to bear on these two inter-related bodies of theory. In the case of the religious prosociality hypothesis, much work has focused on its prediction that religious individuals should behave more prosocially than the non-religious. Self-report measures reliably indicate that religious individuals do indeed *profess* higher levels of various prosocial behaviours than non-religious individuals, including such behaviours as volunteering, charitable giving, sharing and generosity [1–3,10]. But, studies also indicate that religiosity is positively associated with social desirability, suggesting that religious individuals may simply report greater prosocial behaviours because they are more sensitive to being perceived as other-oriented [11]—without, in fact, acting more prosocially. Research eschewing self-report in favour of observable behavioural outcomes generally indicates a nuanced relationship. While some behavioural studies have reported no effect of religiosity, most reviews of the behavioural literature suggest that there is indeed an association of religion with prosociality, but that it is tightly circumscribed by multiple factors [1,2]. These factors include the target's need state [1], the target's overall social distance from the participant [1] and whether the target is an ingroup or an outgroup member [2,3].

The self-report and behavioural studies reviewed above have been offered as support for the cultural evolutionary model, as well as for the religious prosociality hypothesis. But, in the case of the cultural evolutionary model, researchers have also turned to anthropology, archaeology and history for corroborating evidence. Consistent with the cultural evolutionary model, their analyses of ethnographic accounts and the historical record indicate that religion in traditional, small-scale societies is generally characterized by supernatural agents with little interest in human moral affairs, and little ability to influence prosocial behaviour. As societies increase in scale, however, religion has become marked by powerful moralizing agents that actively monitor human affairs and administer rewards and punishments for behaviours regulated by prosocial norms (for review, see [3,5]).

Self-report, behavioural observations and findings from ethnography and history thus offer support for both the religious prosociality hypothesis and the cultural evolutionary model. But, both theories include a crucial claim of causation—that religion (at least when marked by moralizing supernatural agents) actively increases prosocial behaviour. Any causal claim is best supported by experimental evidence; therefore, findings from the experimental literature are particularly important in evaluating these dual bodies of theory. In the case of the religious prosociality hypothesis, correlations of religiosity with either self-reported or observed prosociality might reflect a tendency of more prosocially oriented individuals to adopt religious beliefs and practices, rather than indicating a causal role of religion [1]. Alternatively, a third variable might account for the association—for instance, individuals with a more agreeable personality might tend to be both more prosocial and more religious [1]. In the case of the cultural evolutionary model, conclusions that religious beliefs and practices characterized by Big Gods co-occurred with the emergence of large-scale cooperative societies do not establish that religion is causative [5]. Thus, experimental findings are a vital supplement to the evidence provided by anthropology, history and sociology. Experimental evidence that religion can, and does, promote prosocial behaviour bolsters the cultural evolutionary model and argues against alternative scenarios—particularly the suggestion that the rise of prosocial religions is purely a by-product of societal scale [5].

Experimental evidence that religion increases prosocial behaviour derives largely from studies based on priming [2,12]. In religious priming studies, researchers present a stimulus designed to activate religious cognition, which then (by hypothesis) influences thinking and behaviour in other domains, without participants being consciously aware of the link between stimulus and subsequent behaviour [12]. Multiple methods have been used to prime religion [12]. Subliminal priming [12,13] and implicit priming techniques [14] are designed to minimize conscious awareness of the stimulus. A common implicit priming technique, for example, is to present participants with themed target words embedded in a scrambled sentence task (e.g. [15]). The themed words are presumed to activate the relevant concept (e.g. religion) without participants being overtly aware of it. Explicit techniques, on the other hand, present relevant stimuli in the laboratory without pretence of limiting conscious awareness of the stimulus itself, but in doing so they also increase the likelihood that participants will link awareness of the stimulus to subsequent behaviours of interest to researchers [12] and, in the process, create experimental demand effects [16]. Contextual primes, finally, present primes in natural field settings outside the laboratory; though the stimuli are available to conscious awareness, their presentation may nonetheless be subtle and covert, potentially minimizing demand concerns [12].

The past decade has given rise to a substantial body of work on the behavioural effects of religious priming, including religious priming of prosocial behaviour. Shariff *et al*. [12] recently meta-analysed the full array of religious priming studies as well as religious priming studies devoted to prosociality. Of the 92 experiments they examined, 25 specifically evaluated the effect of religious priming on some measure of prosocial behaviour. For those 25 experiments, Shariff *et al*.'s results indicated that religious priming produced an average effect of *g* = 0.27, 95% CI [0.15, 0.40]. A trim and fill analysis designed to correct this estimate for publication bias [17] reduced the effect size estimate to *g* = 0.18, 95% CI [0.04, 0.32]. An additional meta-analytical tool for identifying publication bias, called the *p*-curve technique [18], likewise suggested the presence of a real effect even after correction for publication bias. Shariff *et al*. [12] also found that religious priming appeared not to affect the behaviour of non-religious participants, contrary to the conclusions of an earlier review [2].

Shariff *et al*.'s meta-analytical findings [12] are important and timely, and have stimulated additional meta-analytical inquiry. van Elk *et al*. [19], for example, meta-analysed Shariff *et al*.'s 2016 data with two other methods that approach the problem of publication bias in different ways. The Bayesian bias correction method [20] produced results that largely accorded with Shariff *et al*.'s original conclusions. Results obtained using the PET-PEESE method [21,22], however, suggested that the population effect size for religious priming did not statistically differ from zero. In the light of these divergent results, van Elk *et al*. [19] argued that large-scale pre-registered replications of influential studies would be necessary to resolve the discrepancies.

One particularly influential study, as van Elk *et al*. also noted [19], is the set of two implicit religious priming experiments conducted by Shariff & Norenzayan [23]. Along with experiments conducted by Pichon *et al*. [13] and Randolph-Seng & Nielsen [24], Shariff and Norenzayan's two experiments are among the earliest examples of religious priming. With 1027 citations as of July 2018 (according to Google Scholar), Shariff and Norenzayan's 2007 paper is by far the most cited work among studies of religious priming and prosociality (versus 296 citations for Pichon *et al*. and 269 for Randolph-Seng and Nielsen, respectively; the median number of citations is 49). Moreover, the implicit priming technique adopted by Shariff & Norenzayan [23]—target words embedded in a scrambled sentence task—is the most commonly employed experimental design in the study of religious prosociality [2].

Because of their considerable influence upon the experimental study of religious prosociality, Shariff and Norenzayan's two studies [23] formed the basis of the current experiments, and we elaborate now upon their methods and results in some detail. In their first experiment, Shariff and Norenzayan presented half of their subjects—those in the religious prime condition—with a set of 10 scrambled sentences, each consisting of five words. To make sense of each set of five words, participants had to disregard one word and rearrange the remaining four words into a meaningful sentence. Half of the scrambled sentences contained a target word intended to prime religious cognition; the other five sentences contained only non-religious words. The second half of the participants were assigned to a control condition in which there was no scrambled sentence task. All participants then took part in a Dictator Game (DG) in the role of giver [25]. Researchers provided subjects with 10 $1 coins and invited them to keep as many coins as they wanted for themselves and to leave as many as they wished for another anonymous player. In a second experiment, Shariff & Norenzayan [23] added two new conditions to the religious prime condition from their first experiment: (i) a neutral prime condition in which participants completed scrambled sentences designed not to evoke any particular concept and (ii) a secular prime condition in which participants completed scrambled sentences containing target words associated with secular moral authority. Participants then completed a DG.

Both of Shariff and Norenzayan's experiments supported the hypothesis that implicit religious priming increases transfers in the DG [23]. In the first experiment, subjects in the religious prime condition gave significantly more money ($4.22 on average) than did unprimed subjects ($1.84 on average). Experiment 2 replicated those results, revealing that subjects in both the religious prime condition and the secular prime condition allocated more money to the other player than did participants in the neutral prime condition. In addition, the researchers found no evidence that the religious priming effect was mediated by conscious awareness of the religious words.

In the wake of recent research questioning the replicability of much psychological research in general [26] and the efficacy of priming studies in particular [27], Gomes & McCullough [28] attempted a direct pre-registered replication of Shariff and Norenzayan's two experiments, using 650 subjects. Gomes & McCullough [28] found no significant difference in DG transfers between subjects in the neutral condition (*M* = $4.49; s.d. = 3.49) and those in the standard religious prime condition (*M* = $4.28; s.d. = 3.67). This failure to replicate Shariff and Norenzayan's 2007 findings led Gomes and McCullough to undertake a meta-analysis of all known studies examining the effect of religious priming on DG transfers. The random-effects meta-analysis of these six experiments implied that the overall effect of religious priming on DG transfers did not statistically differ from zero, although it was in the positive direction, with a medium effect size, *g* = 0.37, s.e. = 0.18, *p* = 0.09, 95% CI [−0.09, 0.83]. The PET-PEESE method suggested a bias-corrected estimate of *g* = −0.12, *p* *=* 0.37, also with a wide 95% CI [−0.45 to 0.21]. The wide confidence intervals associated with both the random-effects estimate and the PET-PEESE estimate, not to mention the small number of experiments that to that date had directly examined the effects of religious priming on generosity in the DG, clearly indicated that additional pre-registered replications are needed, as both Gomes & McCullough [28] and van Elk *et al*. [19] argued.

Shariff & Norenzayan [29] proposed another reason for additional experiments on this topic: Gomes and McCullough's control subjects transferred more of their DG endowments on average (44.9%) than did the subjects in the previous five experiments (14–33% of the endowment). The reasons for this high baseline transfer relative to prior experiments remain unclear, but may include differences between the populations sampled, varying levels of perceived participant anonymity or other differences in methodology [29]. Shariff and Norenzayan suggested that the relatively high baseline levels of generosity among Gomes and McCullough's subjects, whatever their causes, could have attenuated the effect of religious priming. Indeed, in their view, the relatively high levels of baseline generosity indicated that participants in Gomes and McCullough's experiment were already strongly motivated toward generosity. According to Shariff & Norenzayan [29], these relatively high baseline levels of generosity precluded a fair test of their central hypothesis that religious priming downregulates selfishness, and suggest that Gomes and McCullough's experiment addressed the separate question of whether religious priming produces ‘hyperfair’ [29, p. e105] behaviour when prosocial motivation is already high. Future research, they suggested, might fruitfully seek to address both of these questions.

Influential theories—the religious prosociality hypothesis and a cultural evolutionary model of religious prosociality—thus rely importantly, though not exclusively, on experimental evidence largely derived from priming studies. But, divergent meta-analytical findings and disputed interpretations of a failed pre-registered replication attempt leave the priming results open to ongoing scepticism and highlight the need for additional pre-registered replication studies to help clarify the status of the experimental evidence.

### 1.1. The present research

The present research attempted to help address these issues, using two pre-registered replications of Shariff and Norenzayan's 2007 experiments [23]. These pre-registered replications took place in an online environment where participant's anonymity was virtually assured. This methodological adjustment eliminated one of the factors that might encourage hyperfair offers and thus confound efforts to determine whether religious priming increases prosocial behaviour as measured by the DG. In Experiment 1, we used the standard DG, following Shariff and Norenzayan's procedures as closely as possible. In Experiment 2, we introduced a ‘taking’ option to the standard DG, thereby enabling dictators to take money from, as well as give money to, the other participant. The procedure used here followed that of List [30], whose ‘Take $5’ treatment resulted in distributions that appeared considerably more selfish than those obtained under standard DG conditions. This method, implemented in an anonymous online setting, provided an experimental context in which prosocial demand characteristics were lacking, and thus, an environment in which religious priming had ample opportunity to increase prosocial behaviour.

In these two experiments, however, we went beyond simply seeking to replicate Shariff and Norenzayan's 2007 experiments [23]. Most notably, we experimentally evaluated not only Shariff and Norenzayan's implicit priming condition but also a commonly used explicit method for activating religious cognition. For the explicit technique, we had participants write an essay about their beliefs and feelings about God and their religion, similar to methods used by Inzlicht & Tullett [31] and McCullough *et al.* [32]. By exploring two different approaches to religious priming, we sought to determine (i) which major priming methods increase DG transfers and (ii) whether conscious awareness of priming materials may underlie any observed effects of implicit priming on DG transfers.

As we noted earlier, multiple priming methods were available. Here, we focused on implicit and explicit primes, as these are the most common methods used in the literature to date, accounting for more than 82% of the priming studies surveyed by Shariff *et al*. [12]. Beyond variation in method, however, religious primes may differ importantly in content, potentially activating various aspects of religious identity or other psychological mechanisms that could differentially impact prosocial behaviour. For instance, some theorists currently emphasize as a likely mechanism the extent to which individuals view God primarily as punishing and authoritarian, versus benevolent and forgiving [5,8,9,33–35]. Research has yet to resolve this issue, however, and recent meta-analyses (e.g. [12,19,28]) report effect sizes irrespective of putative mechanism.

Because it remains unclear exactly how religious priming produces its effects—if any—we chose our primes accordingly. First, we abjured explicit primes that rely upon reading passages (e.g. [36–38]), as these primes are most likely to vary in terms of what exactly is being primed, and are subject to extensive researcher interpretation. The essay-based explicit prime that we have chosen requires participants to write about their idea of religion or God—whatever that may be. Thus, the essay should have primed whatever aspect of religion was most salient to the participant, rather than an aspect pre-selected by researchers and imperfectly captured (at best) by a representative reading passage. Our essay-based prime was therefore well suited to explicitly prime religion to the same extent as the average explicit priming study included in Shariff *et al*.'s meta-analysis [12], where—again—prosocial effects are reported irrespective of mechanism.

What about the choice of our implicit primes? We note that any implicit religious prime is open to the same charges that can be levied against reading-based explicit primes, namely that the specific target words may be priming specific aspects of religion that are more or less relevant to specific psychological mechanisms identified by theory. Here, however, our decision to use the same implicit primes as Shariff & Norenzayan [23] was justified simply by precedent and the current research context. Our choice followed from the sheer influence of that particular study, as described earlier, and secondly from specific questions arising from Gomes and McCullough's recent failed replication attempt [29].

Although our priming conditions did not enable us to directly assess whether a particular mechanism may be driving any observed effects of religious priming upon prosocial behaviour as measured by the DG, we included an additional measure that enabled us to examine whether a view of God as a punishing versus benevolent agent moderated any effects we found. This measure was the A/B-God scale [39], an instrument that asks participants to rate a series of 18 traits from 1 to 7 according to how much they personally believe each trait accurately characterizes God. The instrument consists of two sub-scales: the Authoritarian (or ‘A’) sub-scale exemplified by such traits as ‘angry’, ‘punishing’ and ‘wrathful’; and the Benevolent (or ‘B’) sub-scale exemplified by such traits as ‘caring’, ‘merciful’ and ‘forgiving’. With this measure, we tested the prediction that the degree to which participants view God as authoritarian moderates the effects of religious priming upon DG transfers.

Our experiments were limited in that they did not examine all variables likely relevant to religious prosociality—including ingroup/outgroup differences and target need state. Nevertheless, published meta-analyses of the effect of religious priming on prosociality that are cited in support of the cultural evolutionary model and the hypothesis of religious prosociality (e.g. [12]), report effects irrespective of target need state and ingroup status (as well as of mechanism). Our studies were intended to help clarify lingering issues with this particular collection of evidence. To be sure, one or two studies cannot decisively adjudicate the status of such a broad body of experimental evidence, marked by diverse methods and multiple outcome variables. We put forth the current experiments in the hope that they will nonetheless constitute a step forward in resolving ongoing uncertainty. In our view, it is best to build up a body of evidence piece by piece, and thus, we focused on the following empirical question—‘does religious priming increase DG transfers?’ It is important to answer this question clearly if theories regarding cultural evolution and religious prosociality are to stand atop a firm empirical foundation.

## 2. General method

### 2.1. Participants

For each experiment, we recruited subjects from Amazon's Mechanical Turk for a 2 (priming condition: religious versus control) × 2 (priming method: explicit versus implicit) × 2 (religiosity: religious versus non-religious) between-subjects design. Our pre-registration specified that we would attempt to arrive at 194 usable subjects (after exclusions) in each of four major groups: (i) subjects given the explicit religious prime; (ii) subjects given the explicit control prime; (iii) subjects given the implicit religious prime and (iv) subjects given the implicit control prime. Subjects were required to have a minimum 90% approval rate on MTurk for previously performed tasks and to reside in the USA. For each subject, we advertised a task offering a modest payment (less than $1.00 guaranteed; see Methods of each experiment for details) for completing a 15–20 min experiment involving one or more decision-making tasks, questionnaires and/or writing tasks. Subjects were also informed that they might receive additional money depending on choices made during the set of decision-making tasks.

A total of 1909 subjects completed the experiments—949 for Experiment 1 and 960 for Experiment 2. Owing to a higher dropout rate in the explicit priming conditions, we ended up with more implicitly primed subjects per cell than expected (approx. 300 per cell before exclusions, rather than 194), but fewer explicitly primed subjects per cell than expected (approx. 176 per cell before exclusions, rather than 194). Subjects were excluded in accordance with pre-registered criteria: we excluded participants whose responses to the suspicion probe indicated suspicion that the study had to do with a link between religion and prosociality, and we excluded participants who demonstrated insufficient attention to the task. Altogether, 218 Experiment 1 subjects (23%) and 192 Experiment 2 subjects (20%) were excluded, largely due to incorrect responses in the implicit priming task and to essays of inadequate length. See electronic supplementary material for details.

### 2.2. Procedures

#### 2.2.1. Overview

After electing to take part in the experiment and providing informed consent, subjects were randomized into one of four conditions: implicit religious priming; implicit priming control; explicit religious priming and explicit priming control.

Subjects completed five basic tasks during the experimental session: (i) the priming task; (ii) the DG; (iii) a suspicion probe; (iv) a demographic questionnaire, and (v) the A/B-God scale [39]. After completing their priming task, subjects were given instructions to the DG and asked to make their DG decision. To guarantee anonymity, subjects were assured that their identity would remain anonymous to the experimenters and to the other subject, and that there would be no contact between subjects. After making their decision in the DG, subjects completed a short questionnaire probing for suspicion, then provided basic demographic data and information on their religious background. Finally, they completed the A/B-God scale [39].

#### 2.2.2. Priming conditions

The *implicit religious priming condition* used scrambled sentences that matched those from the religious prime condition of Study 1 and Study 2 from Shariff & Norenzayan [23]. The *implicit priming control condition* likewise used scrambled sentences identical to those from the control condition of Shariff & Norenzayan, Study 2 [23].

The *explicit religious priming condition* was based on the religious condition of McCullough *et al*., Experiment 1 [32]. Subjects in this condition were asked to write a 5-minute essay about their beliefs and feelings about God and their religion. To make this condition relevant to non-religious subjects, those who were not religious were asked to write about what ‘the idea of God’ means to them. In the *explicit control condition*, subjects were asked to write a 5-minute essay about the sorts of items they have in their home, apartment or dormitory room.

Coding for primes was as follows: 1 for religious prime, 0 for neutral prime; 1 for explicit prime and 0 for implicit prime.

See appendix A for the exact wording of all primes.

#### 2.2.3. Religiosity

A categorical indicator of religiosity distinguished religious subjects (coded as 1) from non-religious subjects (coded as 0). As in Experiment 2 of [23], non-religious subjects were those who identified themselves as either atheist or agnostic, *and* who scored below the midpoint of a 7-point scale assessing belief in God.

#### 2.2.4. Authoritarian view of God

Using participants’ scores on the Authoritarian (A) sub-scale of the A/B-God scale [39], we created a variable that captured the extent to which subjects viewed God as a punishing, authoritarian figure. Scores on this variable were computed as the subject's average endorsement of nine adjectives used to capture God's more punitive characteristics, on a scale of 1 to 7. Such adjectives include ‘angry’, ‘punishing’ and ‘wrathful’. Observed authoritarian scores in Experiment 1 ranged from 1 to 7 (*M* = 3.70, s.d. = 1.36) and did so also in Experiment 2 (*M* = 3.74, s.d. = 1.35).

#### 2.2.5. Manipulation check

To provide a manipulation check for subjects in the explicit priming conditions, we drew upon the Linguistic Inquiry and Word Count 2015 software (LIWC2015: [40]). We used LIWC to calculate the percentage of each essay composed of words in the category of ‘Religion’ (which is pre-defined in the software). Using an independent groups *t*-test with a one-tailed *α* of 0.05, we verified that the average percentage of religious words in essays composed by religiously primed subjects in both Experiment 1 (*M* = 6.24, s.d. = 3.09) and Experiment 2 (*M* = 6.96, s.d. = 2.92) exceeded the average percentage of religious words in essays composed by neutrally primed subjects (Experiment 1 *M* = 0.05, s.d. = 0.33; Experiment 2 *M* = 0.03, s.d. = 0.15; Experiment 1 *t*_88.12_ = 18.70, s.e. = 0.33, *p* < 0.001, Experiment 2 *t*_102.39_ = 24.03, s.e. = 0.29, *p* < 0.001, equal variances not assumed).

### 2.3. Predictions

1A. According to the religious priming hypothesis, there will be a main effect of religious priming, such that the average DG transfer for all participants receiving a religious prime will be greater than the average DG transfer for all participants receiving a neutral prime.
1B. According to the religious priming hypothesis, there will be a simple effect of religious priming, such that the average DG transfer for all participants receiving an implicit religious prime will be greater than the average DG transfer for all participants receiving an implicit neutral prime.
1C. According to the religious priming hypothesis, there will be a simple effect of religious priming such that the average DG transfer for all participants receiving an explicit religious prime will be greater than the average DG transfer for all participants receiving an explicit neutral prime.
2A. According to the religious priming hypothesis as elaborated in [12], there will be a significant two-way interaction such that the main effect of religious priming (regardless of priming method) is greater for religious participants than for non-religious participants, and the effect on non-religious participants will not differ statistically from zero.
2B. According to the religious priming hypothesis, the simple effect of implicit religious priming will be positive and greater for religious participants than for non-religious participants, and the effect on non-religious participants will not differ statistically from zero.
2C. According to the religious priming hypothesis, the simple effect of explicit religious priming will be positive and greater for religious participants than for non-religious participants, and the effect on non-religious participants will not differ statistically from zero.
3. According to the religious priming hypothesis, the effect of implicit religious priming will remain robust to removal from the analysis of subjects who report conscious awareness of religious words during the suspicion probe, as proposed in Gomes & McCullough [28].
4. According to the cultural evolutionary model of religious prosociality, effects of religious priming will be moderated by the extent to which participants view God as authoritarian.

### 2.4. Data analyses

To test these predictions, we constructed three generalized linear models (GLMs) in SPSS V.23 for each experiment, in order to examine the effects of three binary variables—priming condition (religious versus neutral), priming method (explicit versus implicit) and subject religiosity (religious versus non-religious)—on DG transfers. In our first model, GLM #1, we included DG transfer as the dependent variable and priming condition, priming method and religiosity as predictors, along with all two-way and three-way interactions. In our second model, GLM #2, we included DG transfer as the dependent variable and the priming condition, subject religiosity and their interaction as predictors, but only for subjects primed with implicit methods. In our third model, GLM #3, we included as predictors priming condition, subject religiosity and their interaction, but only for subjects primed with explicit methods. In electronic supplementary material, tables S7–S10, we also make available results of exploratory non-parametric tests, which were not pre-registered and which we conducted to be consistent with Gomes & McCullough [28], who noted that the distributions of DG transfers are not optimally suited to the assumptions of general linear models (conclusions were unchanged).

#### 2.4.1. Predictions 1A, 1B and 1C

Prediction 1A (which called for a main effect of religious priming, regardless of priming method) was evaluated on the basis of the significance of priming condition in GLM #1 (one-tailed *α* = 0.05). Prediction 1B (which called for a main effect of implicit religious priming) was evaluated on the basis of the significance of priming condition in GLM #2. Prediction 1C (which specified a main effect of explicit religious priming) was evaluated based on the significance of effect for priming condition in GLM #3 (one-tailed *α* = 0.05).

With 776 total subjects expected per experiment, we estimated greater than 80% power to detect a main effect of priming equal to *d* = 0.18, the bias-corrected estimate of the overall effect of religious priming on prosociality obtained by Shariff *et al*. [12]. With 194 subjects per group for the relevant comparisons, we estimated 90% power to detect a simple effect of implicit or explicit religious priming at *d* = 0.30, or 55% power at *d* = 0.18.

Because our maximum projected sample size for this study fell short of the number of subjects needed to obtain 90% power to detect main and simple effects of *d* = 0.18, the risk of obtaining a null result due to insensitive data was greater than preferred. To address this issue, we pre-registered and adopted the Bayesian approach of Dienes [41]. As Dienes notes, a study with low *a priori* power may end up successfully discriminating between hypotheses; conversely, even high *a priori* power does not guarantee that the collected data will actually be sensitive enough to distinguish between hypotheses [41]. Therefore, it is essential to gauge attained sensitivity and interpret results accordingly. We did so by computing a Bayes factor, using the online software provided by Dienes [41]. The Bayes factor represents the probability of the data under the research hypothesis relative to the probability of the data under the null hypotheses [41]. Although Bayes factors are continuous in nature, we used the conventional criteria to interpret the computed Bayes factor, with a Bayes factor greater than or equal to 3 indicating significant evidence in favour of the research hypothesis (that religious priming increases DG transfers), a Bayes factor less than or equal to 1/3 indicating significant evidence in favour of the null hypothesis (that religious priming does not increase DG transfers) and a Bayes factor between 1/3 and 3 indicating insensitive data [41]. For any non-significant result, using the Bayes factor in this fashion allowed us to determine whether our results provided evidence in favour of the null, or merely indicated insensitive data—a distinction not possible with null hypothesis significance testing, even with high *a priori* power.

To compute a Bayes factor using the tools of Dienes [41], we had to specify a plausibility distribution of effects predicted by the research hypothesis. (We defined the effect of religious priming as the difference between the average DG transfer made by subjects receiving a religious prime and the average DG game transfer made by subjects receiving a neutral prime.) Dienes offers three commonly encountered scenarios, all of which were adapted to the present circumstance: a uniform distribution for the predicted effects; a normal distribution for the predicted effects and a half-normal distribution for the predicted effects [41]. With a uniform distribution, all predicted effects between a minimum and maximum value are assumed to be equally likely under the research hypothesis. In our case, the research hypothesis predicted that religious priming would increase the DG transfers of sufficiently selfish individuals, but not lead to ‘hyperfair’ offers [29, p. 105] greater than 50% of endowment. Thus, we modelled the predicted effect of the research hypothesis as a uniform distribution ranging from 0 (no difference in DG transfers) to (1/2 endowment minus the average DG transfer for neutrally primed subjects). The second distribution of predicted effects suggested by Dienes is a normal distribution, with values centred on a most likely estimate and decreasing in probability as they diverge to either side of that point estimate. Because the research hypothesis predicts both a zero probability of an effect size less than zero and a zero probability of an effect size greater than the difference between half-endowment and the average transfer of neutrally primed subjects, we centred the normal distribution upon the midway point between zero (no difference in DG transfers) and (1/2 endowment minus the average DG transfer for neutrally primed subjects). Following Dienes [41], the standard deviation of this normal distribution was 1/2 of the value of the midpoint, effectively ensuring a zero probability for a predicted effect size of zero and a zero probability for a predicted effect resulting in the transfer of half of the subject's endowment. The third and final distribution of predicted effects suggested by Dienes is the half-normal distribution, which assumes that effects near zero are more likely than effects closer to a maximal value [41]. We modelled the religious priming hypothesis using the half-normal distribution as follows: the modal effect size of the predicted distribution was set at zero. As predicted effect sizes increase above zero, they become increasingly less likely, until they reach zero probability when the effect results in a transfer of 1/2 the subject's endowment—the maximal value. Following Dienes [41], to achieve this distribution of predicted effects, we set the standard deviation of this half-normal distribution equal to 1/2 the difference between zero (no difference in DG transfers) and (1/2 endowment minus the average DG transfer for neutrally primed subjects). To ensure that our results were robust to differences in modelling the predicted effects, we report Bayes factors computed with the three approaches detailed above.

#### 2.4.2. Predictions 2A, 2B and 2C

Prediction 2A (which called for the main effect of religious priming across all priming methods to be positive among religious participants, but zero among non-religious participants) was evaluated on the basis of the significance of the interaction between priming condition and subject religiosity in GLM #1. Prediction 2B (which called for the main effect of implicit religious priming to be positive among religious participants, but zero among non-religious participants) was evaluated on the basis of the significance of the interaction between priming condition and subject religiosity in GLM #2. Prediction 2C (which called for the main effect of explicit priming to be positive among religious participants, but zero among non-religious participants) was evaluated on the basis of the significance of the interaction between priming condition and subject religiosity in GLM #3. All of these evaluations were performed using a one-tailed *α* of 0.05.

Because power was less than 90% to detect the interaction, we again used the Bayesian methods of Dienes [41] to interpret results. We defined the interaction effect as the effect of a religious prime upon religious subjects (*d*_r_) minus the effect of a religious prime upon non-religious subjects (*d*_n_). Interpreted conservatively, the research hypothesis predicts that the effect of a religious prime upon religious subjects will exceed its effect upon non-religious subjects (*d*_r_ > *d*_n_). On the research hypothesis, the range of values for the interaction effect thus runs from 0 (when *d*_n_ = *d*_r_) to *d*_r_ (when *d*_n_ = 0). Following the example of Dienes [41], we therefore pre-registered that we would use a uniform distribution ranging from 0 to *d*_r_ to model the plausibility distribution of the interaction effect predicted by the research hypothesis. And we again used the conventional criteria to interpret the resulting Bayes factor for this interaction [41].

We note here, however, that subsequent Bayesian analyses of interaction effects revealed limitations with our pre-registered analytical strategy for modelling the predictions of the priming hypothesis, at least where interactions were concerned. Specifically, our pre-registered strategy defined the plausibility distribution of the interaction effect by using a uniform distribution that ranged from a lower bound of zero to an upper bound equal to the priming effect that was actually observed among religious subjects. This approach assumed—consistent with the priming hypothesis—that this effect would be positive, but subsequent analyses involving implicit priming revealed that assumption to be flawed. In cases where the priming effect among religious subjects was negative, our procedure yielded a Bayes factor of 0.00, indicating that the data were literally impossible under the research hypothesis.

Such a result led us to seek a more appropriate method for modelling the plausibility distribution of the interaction effect, one in which the upper bound of the plausibility distribution would always be positive and would more realistically reflect the predictions of the priming hypothesis. It seemed to us that the most straightforward post hoc approach was to take full advantage of a clear prediction made in recent formulations of the priming hypothesis—namely, that religious priming has no effect on non-religious subjects. If that is the case, then the interaction effect should be equal to the possible effect of religious priming among religious subjects only. This possible effect, in turn, would best be modelled as a uniform distribution with a lower bound of 0 (reflecting no difference in DG transfers between religiously primed and neutrally primed religious subjects) and an upper bound equal to the maximum *expected* size (rather than observed size) of the priming effect among religious subjects. For Experiment 1, this difference would be the difference between 50 cents (0.50) and the mean DG transfer of neutrally primed religious subjects. For Experiment 2, this difference would be the difference between 25 cents (0.25) and the mean DG transfer of neutrally primed religious subjects. In view of the problems with our pre-registered strategy for analysing the interaction effect, we report in the main text only these post hoc Bayesian analyses of the interaction, along with Bayesian analyses of the simple effects of priming at the levels of both religious and non-religious subjects. However, we also provide results of our pre-registered Bayesian analyses of the interaction in the appropriate summary tables (tables 2 and 5; electronic supplementary material, tables S2 and S5).

#### 2.4.3. Prediction 3

We pre-registered that we would test Prediction 3 (which called for the effect of implicit religious priming to be robust to the removal of subjects who report conscious awareness of religious words during the suspicion probe) only if Prediction 1B or Prediction 2B was supported. Because neither Prediction 1B nor Prediction 2B was supported, we performed no analyses bearing on Prediction 3.

#### 2.4.4. Prediction 4

To test Prediction 4, we conducted moderation analyses using the A/B-God measure [39], restricting our sample to Christian respondents because the measure has been validated using only a Christian sample. Using this restricted sample, we constructed three GLMs, each of which regressed DG transfers on priming condition, with subjects' scores on the Authoritarian (A) sub-scale entered as a moderator. There were three such analyses—one for all Christian subjects (GLM #4), one for Christian subjects receiving an implicit prime (GLM #5) and one for Christian subjects receiving an explicit prime (GLM #6). Results were evaluated based on the significance of the interaction between priming condition and Authoritarian score in the corresponding GLM. All of these evaluations were performed using a one-tailed *α* of 0.05.

We had planned, in the case of null results, to employ the Bayesian methods of Dienes [41], as follows. First, we specified the two levels of score on the Authoritarian sub-scale at which to compare the effects. We defined the ‘High’ level of Authoritarian score as one standard deviation above the mean, and we defined the ‘Low’ level of Authoritarian score as one standard deviation below the mean. We then defined the interaction effect as the effect of a religious prime at the level of ‘High’ Authoritarian score (*d*_H_) minus the effect of a religious prime at the level of ‘Low’ Authoritarian score (*d*_L_).

The research hypothesis predicts that the effect of a religious prime at the ‘High’ level of Authoritarian score will be greater than that of a religious prime at the ‘Low’ level of Authoritarian score (*d*_H_ > *d*_L_). On the research hypothesis, the range of values for the interaction effect thus runs from 0 (when *d*_H_ = *d*_L_) to d_H_ (when *d*_L_ = 0). Following the example of Dienes [41], we therefore pre-registered that we would use a uniform distribution ranging from 0 to *d*_H_ to model the plausibility distribution of the interaction effect predicted by the religious priming hypothesis, and we used the previously described conventional criteria to interpret the resulting Bayes factor for this interaction.

We note here some shortcomings of our pre-registered strategy. First, in requiring us to split the sample into two groups based on authoritarian views of God, and then to compare the effects of religious priming across those two groups, our pre-registered strategy entailed that we eliminate a substantial proportion of subjects from consideration and evaluate our primary analysis on the basis of a second, less powerful analysis. Our pre-registered Bayesian analytical approach was also challenged by how we specified the plausibility distribution of the interaction effect predicted by the priming hypothesis. This is the same issue we encountered when describing the data analyses for Predictions 2A–2C: our pre-registered strategy assumed that DG transfers among religiously primed subjects would be greater than transfers among neutrally primed subjects, and that this positive difference would then form the upper bound for the uniform distribution used to model the predicted interaction effect. For implicitly primed subjects, however, mean DG transfers were *lower* among religiously primed subjects than among neutrally primed subjects, rendering our planned strategy arguably problematic in that the upper bound of the uniform distribution was lower than the lower bound, returning a Bayes factor of 0.00. To address this concern, we conducted an additional set of non-pre-registered Bayesian analyses. In this alternative approach, and similar to what we described above for Predictions 2A–2C, we modelled the upper bound of the plausibility distribution of the interaction effect as the expected rather than the observed range of the religious priming effect among subjects with a highly authoritarian view of God—a value that would always be positive. For Experiment 1, this upper bound was $0.50 minus the average DG transfer among neutrally primed High Authoritarians. For Experiment 2, this upper bound was $0.25 minus the average DG transfer among neutrally primed High Authoritarians. Given the issues with our pre-registered strategy for analysing the interaction effect, we address in the main text only these post hoc Bayesian analyses of the interaction. However, we do make available the results of our pre-registered Bayesian analyses of the interaction (in tables 3 and 6; electronic supplementary material, tables S3 and S6).

*Pre-registration*. After this replication proposal was accepted but before any data were collected, we pre-registered both experiments on the Open Science Framework (https://osf.io/6nqwt/).

## 3. Overview of key findings

To help readers navigate our rather extensive results, below we provide a brief summary of essential findings from the experiments, and we direct readers to the locations of the relevant supporting evidence.

The key findings are as follows:

(1) *Religious participants transferred more money in the DGs than did non-religious participants.* (For support, see the subsections entitled ‘Did religious subjects transfer more money than did non-religious subjects?’ in the Results section of both Experiment 1 and Experiment 2 (§§4.4 and 5.5).)
(2) *Implicit religious priming did not increase DG transfers among either religious or non-religious participants.* (For support, see the subsections entitled ‘Prediction 2B’ in the Results section of both Experiment 1 and Experiment 2 (§§4.9 and 5.10).)
(3) *A new meta-analysis of all available studies reinforces the conclusion that implicit religious priming does not increase DG transfers.* (For support, see the Study 3 Results section (§6.1)).
(4) *Explicit religious priming did not appear to increase* DG *transfers among non-religious participants, but may have increased transfers among religious participants, with a small effect size.* (For support, see the subsections entitled ‘Prediction 2C’ in the Results section of both Experiment 1 and Experiment 2, as well as the subsection of the General discussion entitled ‘Implicit versus explicit religious priming' (§§4.10, 5.11 and 7.2).)

## 4. Experiment 1

### 4.1. Subjects

The mean age of the sample was 35.54 years (s.d. = 11.50). A sizable majority of participants (80.0%) identified as White, 54.6% of participants were female and 38.4% of participants characterized themselves as either atheist or agnostic.

### 4.2. Procedures

In Experiment 1, subjects took part in a standard DG. We offered each subject a payment of $0.30 plus $0.50 in bonus money. This $0.80 payment was in addition to money that they kept based on their DG decision. Subjects were told that they would be the ‘giver’ in an economic decision-making task, were provided a $1.00 endowment and were asked how much of the endowment, if any, they wished to transfer to a second, anonymous subject. Our DG instructions followed the procedure of Shariff and Norenzayan's 2007 experiments as closely as possible, while adapting the instructions to the online environment as necessary and slightly modifying the wording so as not to deceive subjects. See appendix B for a detailed comparison of the DG instructions and questionnaire items used in this experiment versus those used by Shariff & Norenzayan [23].

### 4.3. Results

Descriptive statistics appear in table 1; means reported in the text are estimated marginal means. A summary of all Experiment 1 results appears in tables 2 and 3. In the main text, we report only the results obtained using the pre-registered exclusion criteria, but we note where conclusions differ if analyses are conducted without excluding any subjects (see electronic supplementary material, table S2 for specifics). Where indicated, *p*-values have been adjusted to reflect one-tailed hypothesis tests. For effect sizes involving mean differences, we report Hedges' *g*. We standardized *g* by using the overall error term from the relevant linear model, and we corrected for bias using the procedure recommended by Borenstein *et al.* [42]. We report *R*^2^_change_ as the effect size for analyses testing whether the effect of religious priming is moderated by the extent to which subjects view God as a punishing, authoritarian figure.

**Table 1.** Descriptive statistics for DG transfers in Experiment 1.

priming method	religiosity	priming condition mean ± s.d. (*N*)		
		control	religious priming	total
explicit	non-religious	0.219 ± 0.252 (48)	0.270 ± 0.304 (33)	0.240 ± 0.273 (81)
	religious	0.319 ± 0.268 (104)	0.378 ± 0.343 (55)	0.340 ± 0.296 (159)
	total	0.288 ± 0.266 (152)	0.338 ± 0.331 (88)	0.306 ± 0.292 (240)
implicit	non-religious	0.238 ± 0.274 (90)	0.228 ± 0.291 (83)	0.233 ± 0.281 (173)
	religious	0.309 ± 0.267 (160)	0.290 ± 0.286 (147)	0.300 ± 0.276 (316)
	total	0.284 ± 0.271 (259)	0.267 ± 0.289 (230)	0.276 ± 0.279 (489)
total	non-religious	0.231 ± 0.266 (138)	0.240 ± 0.294 (116)	0.235 ± 0.278 (254)
	religious	0.313 ± 0.267 (273)	0.314 ± 0.305 (202)	0.313 ± 0.283 (475)
	total	0.285 ± 0.269 (411)	0.287 ± 0.302 (318)	0.286 ± 0.284 (729)

**Table 2.** Experiment 1 results summary: priming effects and interactions with religiosity.

prediction	model	mean difference	s.e.	*p-*values^a^	n1 prime	n2 control	*g*	s.e. of *g*	Bayes uniform	Bayes normal	Bayes 1/2 normal
1A: priming effect, all methods and subjects	GLM #1	0.020	0.024	0.199	318	411	0.07	0.07	0.30	0.17	0.45
1B: implicit priming effect, all subjects	GLM #2	−0.015	0.026	0.709	230	259	−0.05	0.09	0.10	0.06	0.15
1C: explicit priming effect, all subjects	GLM #3	0.055	0.041	0.088	88	152	0.19	0.13	1.00	0.99	1.34
2A: interaction with religiosity, all methods	GLM #1	−0.0005	0.047	0.504	318	411	—	—	0.97^b^	n.a.	n.a.
2A: interaction with religiosity, all methods	GLM #1	−0.0005	0.047	0.504	318	411	—	—	0.32^c^	n.a.	n.a.
2A: priming effect, all methods, religious subs only	GLM #1	0.020	0.028	0.241	202	273	0.07	0.09	0.37	0.27	0.55
2A: Priming effect, all methods, non-religious subs	GLM #1	0.020	0.038	0.298	116	138	0.07	0.13	0.28	0.18	0.43
2B: interaction with religiosity, implicit priming only	GLM #2	−0.009	0.053	0.568	230	259	—	—	0.00^d^	n.a.	n.a.
2B: interaction with religiosity, implicit priming only	GLM #2	−0.009	0.053	0.568	230	259	—	—	0.30^c^	n.a.	n.a
2B: implicit priming effect, religious subs only	GLM #2	−0.019	0.031	0.728	147	169	−0.07	0.11	0.13	0.09	0.21
2B: implicit priming effect, non-religious subs only	GLM #2	−0.010	0.042	0.594	83	90	−0.04	0.15	0.17	0.11	0.26
2C: interaction with religiosity, explicit priming only	GLM #3	0.008	0.080	0.461	88	152	—	—	0.95^b^	n.a.	n.a.
2C: interaction with religiosity, explicit priming only	GLM #3	0.008	0.080	0.461	88	152	—	—	0.58^c^	n.a.	n.a.
2C: explicit priming effect, religious subs only	GLM #3	0.059	0.048	0.111	55	104	0.20	0.17	1.25	1.38	1.46
2C: explicit priming effect, non-religious subs only	GLM #3	0.051	0.065	0.218	33	48	0.18	0.23	0.62	0.60	0.83

^a^*p*-values are one-tailed.

^b^Bayes factor reflects original pre-registered analytical strategy.

^c^Bayes factor reflects revised analytical strategy.

^d^Bayes factor reflects original pre-registered analytical strategy, but the religious priming effect was in the direction counter to theory, rendering computation of the Bayes factor using pre-registered methods problematic.

**Table 3.** Experiment 1 results summary: interactions with view of God as authoritarian figure.

prediction	model	mean difference	s.e.	*p*-value^a^	Bayes factor uniform
‘A’ scale moderates priming effect, all methods	GLM #4	0.032	0.024	0.093	1.08^b^
‘A’ scale moderates priming effect, all methods	GLM #4	0.032	0.024	0.093	2.02^c^
‘A’ scale moderates priming effect, implicit priming only	GLM #5	0.037	0.030	0.104	0.00^d^
‘A’ scale moderates priming effect, implicit priming only	GLM #5	0.037	0.030	0.104	3.14^c^
‘A’ scale moderates priming effect, explicit priming only	GLM #6	0.049	0.043	0.128	1.36^b^
‘A’ scale moderates priming effect, explicit priming only	GLM #6	0.049	0.043	0.128	1.39^c^

^a^*p*-values are one-tailed.

^b^Bayes factor calculated from High/Low split.

^c^Bayes factor calculated from High/Low split; non-pre-registered analytical approach.

^d^Bayes factor calculated from High/Low split; the priming effect among subjects with highly authoritarian views of God was in the direction counter to theory, rendering computation of the Bayes factor using pre-registered methods problematic.

### 4.4. Did religious subjects transfer more money than did non-religious subjects?

Yes. Our results focus on the effects of priming condition and on possible interactions of priming condition with subject religiosity. We would be remiss, however, if we failed to mention at the outset that we observed a persistent main effect of subject religiosity in Experiment 1, evident among implicitly primed subjects, *F*_1,485_ = 6.39, *p* = 0.012 (two-tailed; GLM #2); among explicitly primed subjects, *F*_1,236_ = 6.64, *p* = 0.011 (two-tailed; GLM #3) and among all subjects considered regardless of priming method, *F*_1,721_ = 12.85, *p* < 0.001 (two-tailed; GLM #1). Across all subjects, religious subjects transferred 8.6 cents more on average than did non-religious subjects, *g* = 0.30 95% CI [0.15, 0.46].

### 4.5. Prediction 1A: Was there a main effect of religious priming, such that the average Dictator Game transfer for all subjects receiving a religious prime (regardless of priming method) exceeded the average Dictator Game transfer for all subjects receiving a neutral prime?

No. Results from GLM #1 indicated that the DG transfers of religiously primed subjects (*M* = $0.291) did not significantly differ from those of subjects who received a neutral prime (*M* = $0.271), *t*_721_ = 0.846, s.e. = 0.024, *p* = 0.199 (one-tailed), *g* = 0.07, 95% CI [−0.07, 0.22].

Bayesian analyses of the non-significant finding generally favoured the null hypothesis, but conclusions resulting from the Bayes factors depended upon how the predictions of the priming hypothesis were modelled. When computed using uniform and normal distributions for the predicted priming effect, the Bayes factors were 0.30 and 0.17, respectively, offering reasonable evidence for the null. But, the data did not provide reasonable evidence for the null hypothesis when we used a half-normal distribution to model the expected effect (Bayes factor = 0.45).

### 4.6. Prediction 1B: Was there a simple effect of implicit religious priming, such that the average Dictator Game transfer for all subjects receiving an implicit religious prime exceeded the average Dictator Game transfer for all subjects receiving an implicit neutral prime?

No. GLM #2 indicated that the DG transfers of subjects who received an implicit religious prime (*M* = $0.259) were not significantly greater than those of subjects who received an implicit neutral prime (*M* = $0.273), *t*_485_ = −0.553, s.e. = 0.026, *p* = 0.709 (one-tailed), *g* = −0.05, 95% CI [−0.23, 0.12]. Indeed, inspection of both the estimated marginal means and the descriptive means indicated that, if anything, subjects receiving an implicit religious prime transferred *less* money than did those receiving an implicit neutral prime.

Bayesian analyses of the non-significant finding supported the null hypothesis regardless of how the predictions were modelled, with Bayes factors ranging from 0.06 (normal distribution) to 0.15 (half-normal distribution). These findings constitute evidence that implicit religious priming did not increase DG transfers, regardless of subjects’ religiosity.

### 4.7. Prediction 1C: Was there a simple effect of explicit religious priming such that the average Dictator Game transfer for all subjects receiving an explicit religious prime exceeded the average Dictator Game transfer for all subjects receiving an explicit neutral prime?

No. GLM #3 indicated that the DG transfers of subjects who received an explicit religious prime (*M* = $0.324) were not significantly greater than those of subjects who received an explicit neutral prime (*M* = $0.269), although the results approached significance using the one-tailed test, *t*_236_ = 1.355, s.e. = 0.041, *p* = 0.088 (one-tailed), *g* = 0.19, 95% CI [−0.07, 0.45].

Consistent with the marginally significant results revealed by the *t-*test, Bayesian analyses indicated that the data did not distinguish the null from the priming hypothesis, with Bayes factors ranging from 0.99 (normal distribution) to 1.34 (half-normal distribution). It thus remains unclear whether explicit priming influenced DG transfers in Experiment 1, considering subjects irrespective of their religiosity.

### 4.8. Prediction 2A: Was there a significant two-way interaction such that the main effect of religious priming (regardless of priming method) was greater for religious subjects than for non-religious subjects, and the effect on non-religious subjects did not differ statistically from zero?

No. Results from GLM #1 indicated that the interaction of priming condition with subject religiosity was not significant, *t*_721_ = −0.01, s.e. = 0.047, *p* = 0.504 (one-tailed). There was no significant effect of religious priming, either for religious subjects (*t*_721_ = 0.71, s.e. = 0.028, *p* = 0.241 (one-tailed), *g* = 0.07, 95% CI [−0.11, 0.25]) or for non-religious subjects (*t*_721_ = 0.53, s.e. = 0.038, *p* = 0.298 (one-tailed), *g* = 0.07, 95% CI [−0.18, 0.32]).

Using our revised approach to analyse interaction effects (see Data analyses), we obtained a Bayes factor for the interaction of 0.32, slightly favouring the null hypothesis that there is no interaction. For the simple effect of priming on specifically *religious* subjects, Bayesian analyses did not provide clear evidence against the priming effect, with Bayes factors ranging from 0.27 (normal distribution) to 0.55 (half-normal distribution). Similarly, Bayesian analyses of the simple effect of priming on specifically *non-religious* subjects failed to furnish clear evidence against the priming effect, with Bayes factors ranging from 0.18 (normal distribution) to 0.43 (half-normal distribution).

### 4.9. Prediction 2B: Among implicitly primed subjects only, was there a significant interaction between priming condition and religiosity, such that the simple effect of implicit religious priming was positive and greater for religious subjects than for non-religious subjects, and that the effect on non-religious subjects did not differ statistically from zero?

No. The interaction of priming condition with subject religiosity was not significant in GLM #2, *t*_485_ = −0.171, s.e. = 0.053, *p* = 0.568 (one-tailed). There was no significant effect of implicit religious priming, either for religious subjects (*t*_485_ = −0.61, s.e. = 0.031, *p* = 0.728 (one-tailed), *g* = −0.07, 95% CI [−0.29, 0.15]), or for non-religious subjects (*t*_485_ = −0.24, s.e. = 0.042, *p* = 0.594 (one-tailed), *g* = −0.04, 95% CI [−0.33, 0.26]).

Using the revised approach to conduct Bayesian analyses of this interaction (detailed in Data Analyses), we computed a Bayes factor of 0.30, slightly favouring the null hypothesis of no interaction (though this conclusion is perhaps qualified by the fact that the Bayes factor was 0.40 when we analysed the full sample, with no exclusions). When we turned to the simple effect of implicit religious priming among specifically *religious* subjects, Bayesian analyses provided evidence that implicit religious priming did *not* affect DG transfers, with Bayes factors ranging from 0.09 (normal distribution) to 0.21 (half-normal distribution). For *non-religious* subjects, Bayesian analyses likewise furnished clear evidence that implicit religious priming had no effect, with Bayes factors ranging from 0.11 (normal distribution) to 0.26 (half-normal distribution). These Bayesian analyses thus indicate that the effect of implicit religious priming did not differ based on the religiosity of the subjects, *and* that the priming effect was zero rather than positive for both non-religious and religious subjects.

### 4.10. Prediction 2C: Among explicitly primed subjects only, was there a significant interaction between priming condition and religiosity, such that the simple effect of explicit religious priming was positive and greater for religious subjects than for non-religious subjects, and that the effect on non-religious subjects did not differ statistically from zero?

No. In GLM #3, the interaction of priming condition with subject religiosity was not significant, *t*_236_ = 0.099, s.e. = 0.081, *p* = 0.461 (one-tailed). There was no significant effect of explicit religious priming either for religious subjects (*t*_236_ = 1.23, s.e. = 0.048, *p* = 0.111 (one-tailed), *g* = 0.20, 95% CI [−0.12, 0.53]) or for non-religious subjects (*t*_236_ = 0.79, s.e. = 0.065, *p* = 0.218 (one-tailed), *g* = 0.18, 95% CI [−0.27, 0.62]).

For the interaction, Bayesian analyses conducted using our revised methods (see Data analyses) were unable to distinguish the null hypothesis from the priming hypothesis, yielding a Bayes factor equal to 0.58, indicating insensitive data. When we examined the simple effect of explicit priming on specifically *religious* subjects, Bayesian analyses did not provide reliable evidence in favour of the null hypothesis, with Bayes factors ranging from 1.25 (uniform distribution) to 1.46 (half-normal distribution). Similarly, Bayesian analyses of the simple effect of explicit priming on specifically *non-religious* subjects failed to furnish reliable evidence in favour of the null hypothesis, with Bayes factors ranging from 0.60 (normal distribution) to 0.83 (half-normal distribution). The data thus did not arbitrate against an effect of *explicit* religious priming, particularly for religious subjects, nor did the data suggest that the effect of explicit priming does not differ between religious and non-religious subjects.

### 4.11. Prediction 3: Did the effect of implicit religious priming remain significant even after removing from the analysis any subjects who reported conscious awareness of religious words during the suspicion probe?

Because there were no significant effects of implicit religious priming, we did not perform this analysis.

### 4.12. Prediction 4: Were the effects of religious priming moderated by the extent to which subjects viewed God as authoritarian?

After exclusions for suspicion, essay length and inattention to the task, 325 subjects who identified as Christian remained in the Experiment 1 sample. These 325 subjects formed the basis for the following moderation analyses, as stipulated in the pre-registration. To test for moderation of the religious priming effect, we regressed DG transfers on experimental condition, subject's authoritarian view of God and the interaction of the two.

*GLM #4: Results for all Christian subjects, regardless of priming method.* When we regressed DG transfers on priming condition, subject's authoritarian view of God and the interaction of the two, the interaction was not significant *t*_321_ = 1.33, s.e. = 0.024, *p* = 0.093 (one-tailed), *R*^2^_change_ = 0.005.

To conduct our Bayesian analyses, we first split the sample of Christian subjects into two groups based on authoritarian views of God, as described previously. After we did so, 113 subjects remained, with individual cell sizes ranging from 22 (for religiously primed subjects with highly authoritarian views of God) to 40 (for neutrally primed subjects with low authoritarian views of God). Using this subsample, we regressed DG transfers on priming condition, the grouping variable that captured authoritarian views of God (High versus Low), and the interaction of the two. There was again no significant interaction between authoritarian views of God and priming condition, *t*_109_ = 0.140, s.e. = 0.024, *p* = 0.083 (one-tailed). Bayesian analyses of this non-significant interaction (using our revised analytical strategy—see Data analyses) returned a Bayes factor of 2.02.

*GLM #5: Results for implicitly primed Christian subjects.* We then tested for moderation of the religious priming effect using only implicitly primed Christian subjects (*N* = 209). We again regressed DG transfers on priming condition, subject's authoritarian view of God and the interaction of the two. The interaction was not significant, *t*_205_ = 1.26, s.e. = 0.030, *p* = 0.104 (one-tailed), *R*^2^_change_ = 0.008.

To conduct Bayesian analyses of this non-significant result, we again split the sample of Christian subjects into two groups based on authoritarian views of God. After we did so, 66 subjects remained, with individual cell sizes ranging from 11 (for neutrally primed subjects with highly authoritarian views of God) to 21 (for neutrally primed subjects with low authoritarian views of God). Using this subsample to regress DG transfers on priming condition, the grouping variable that captured authoritarian views of God (High versus Low), and the interaction of the two, we found a significant interaction between authoritarian views of God and priming condition, *t*_62_ = 1.70, s.e. = 0.126, *p* = 0.048 (one-tailed). Using our alternative approach to specify the plausibility distribution of the interaction effect predicting by the priming hypothesis (see Data analyses), we obtained a Bayes factor of 3.14.

This latter Bayes factor provides evidence that the effect of implicit religious priming varies between subjects who view God as highly authoritarian versus those who view God as low in authoritarianism. In probing the significant interaction, we found that the interaction appears to be driven largely by subjects who view God as low in authoritarianism (Low Authoritarians). The average transfer of neutrally primed Low Authoritarians (*M* = 0.419) was 23.1 cents greater than that of Low Authoritarians who had been religiously primed (*M* = 0.188), *p* = 0.006, two-tailed. The direction of the implicit religious priming effect among Low Authoritarians seems to us contrary to the predictions of the religious priming hypothesis. Religiously primed High Authoritarians (*M* = 0.165) transferred 1.2 cents less than neutrally primed High Authoritarians (*M* = 0.182), a difference that was not statistically significant, *p* = 0.860, two-tailed. Whatever this interaction may represent, it therefore does not appear to indicate that implicit religious priming causes subjects who view God as strongly authoritarian to transfer more money to recipients, while having less of an effect on subjects who view God as weakly authoritarian. Instead, if anything, the interaction would suggest that implicit religious priming causes subjects who view God as low in authoritarianism to give *less* money to recipients, while having little or no effect on subjects who view God as high in authoritarianism.

*GLM #6: Results for explicitly primed Christian subjects.* When we tested for moderation of the religious priming effect using only explicitly primed Christian subjects (*N* = 116), our regression of priming condition, authoritarian view of God and the interaction of the two yielded a non-significant interaction, *t*_112_ = 1.14, s.e. = 0.043, *p* = 0.128 (one-tailed), *R*^2^_change_ = 0.011.

To interpret this null result using Bayesian methods, we split the sample of Christian subjects into two groups based on authoritarian views of God. This resulted in a subsample of 47 subjects, with individual cell sizes ranging from 5 (for religiously primed subjects with highly authoritarian views of God) to 19 (for neutrally primed subjects with low authoritarian views of God). When we regressed DG transfers on priming condition, the grouping variable that captured authoritarian views of God (High versus Low), and the interaction of the two, we found that the interaction was not significant, *t*_43_ = 0.923, s.e. = 0.193, *p* = 0.181 (one-tailed). Bayesian analyses using our revised analytical strategy (see Data analyses) indicated that the data were insensitive rather than supportive of the null (Bayes factor = 1.39), an unsurprising outcome given the small sample size.

Assessing all of the moderation analyses for Experiment 1 collectively, we thus find that the data—despite multiple non-significant results—do not argue against the possibility that the effect of religious priming among Christian subjects varies as a function of how authoritarian the subject understands God to be.

### 4.13. Discussion

Experiment 1 tested the hypothesis that religious priming increases DG transfers, using the standard DG paradigm and two common approaches to implicit and explicit religious priming. We consistently obtained non-significant results, which we interpreted using Bayesian analyses in order to determine whether the data actually supported the null hypothesis, or were merely insensitive. When we collapsed results across the priming method and considered subjects without regard to their religiosity, we found that the data tended to favour the null hypothesis, but were ultimately inconclusive: results were not robust to how we modelled the expected distribution of the priming effect.

Breaking down the results by priming method, however, yielded a clearer picture. We found strong evidence that *implicit* priming did *not* increase DG transfers, considering subjects without regard to their religiosity. Indeed, the implicit priming data of Experiment 1 were anywhere from 6 to 16 times more likely under the null hypothesis than under the research hypothesis. Furthermore, Experiment 1 supported the null hypothesis among both religious *and* non-religious subjects who were implicitly primed, and provided evidence that the effect of implicit priming does not vary based on subject religiosity. The same cannot be said for *explicit* priming, where the data failed to provide a decisive outcome, despite multiple non-significant results. Our results therefore do not arbitrate against the possibility that *explicit* religious priming increases DG transfers, particularly among religious individuals, with a small effect size (the point estimate for explicit priming among religious individuals was 0.20).

In Experiment 1, we had the additional goal of testing whether the effect of religious priming (among Christian subjects only) was moderated by the extent to which subjects viewed God as a punishing, authoritarian figure. Although we obtained non-significant results, Bayesian analyses indicated that we did not have meaningful evidence in favour of the null hypothesis. Experiment 1 therefore offered no evidence against the possibility that the effect of religious priming among Christian subjects varies as a function of how punishing or authoritarian they understand God to be.

## 5. Experiment 2

Experiment 1 relied upon the standard DG paradigm. Because prior theorizing suggested that religious priming might increase DG transfers only when baseline levels of selfishness are sufficiently high [29], Experiment 2 featured a modified DG that allowed dictators the option of taking money *from* recipients, as well as transferring money to them. In other respects, the procedures were essentially identical to those of Experiment 2.

### 5.1. Subjects

Subject payment was identical to Experiment 1, save that the bonus amount was $0.45 rather than $0.50.

The mean age of the sample was 36.39 years (s.d. = 11.95). A sizable majority of participants (80.3%) identified as White, 53.5% of participants were female and 38.2% of participants characterized themselves as either atheist or agnostic.

### 5.2. Procedures

Experiment 2 paralleled Experiment 1 quite closely, varying only in the nature of the DG used. Rather than the standard DG, Experiment 2 relied upon a modified DG with a ‘take’ option, as in the ‘Take $5’ treatment of List [30]. Each dictator was informed that he or she was being paired with another anonymous subject on MTurk, and that both subjects had been endowed with $0.50. Dictators were further informed that they, but not the other subject, had been provisionally endowed with an additional $0.50. Dictators were then asked what portion of this provisional endowment, if any, they wished to transfer to the other subject. Dictators were told that they can also transfer a ‘negative amount—i.e. you can take up to $0.50 from the other subject’. Transfers could occur in increments of $0.10, and thus ranged from −$0.50 (i.e. $0.50 taken from the other player) to $0.50 (i.e. $0.50 given to the other player). See appendix C for the exact wording of the DG procedure in Experiment 2.

### 5.3. Hypotheses and data analyses

All of the hypotheses and analyses detailed for Study 1 also apply to Study 2.

We expected in general that the average DG transfer for each condition in Study 2 would be less than the corresponding DG transfer in Study 1, relative to half-endowment. However, the between-study comparison of DG transfers was not of intrinsic theoretical interest and thus was not subjected to systematic statistical analysis. One scenario, however, merits special note. According to proponents of the religious priming hypothesis, religious primes increase prosociality and thus DG transfers only when baseline levels of participant generosity are sufficiently low. Results from Gomes & McCullough [28] can be interpreted to suggest that in some circumstances, baseline generosity levels may be too high for religious priming to work. If relatively high levels of DG transfers emerged in the neutral prime conditions of Study 1 (approx. 44% of endowment versus 14–33% of the endowment), null results might again be attributed to violations of this hypothesized boundary condition. We included Study 2, with its modified DG paradigm, primarily to address this possibility. Even if Study 1 yielded baseline levels of generosity deemed too high for religious priming to work, the modified paradigm of Study 2 was likely—given the results of List [30]—to produce lower baseline transfers, and thus, it might still enable a test of whether religious priming increases DG transfers under conditions that fall within boundaries acknowledged as appropriate by proponents of religious priming.

### 5.4. Results

Descriptive statistics for Experiment 2 appear in table 4; means reported in the text are estimated marginal means. A summary of Study 2 results appears in tables 5 and 6. In the main text, we report only the results obtained using the pre-registered exclusion criteria, but we note whether conclusions differ when all subjects are included in the sample (see electronic supplementary material, tables S5 and S6 for a summary of Study 2 results using all subjects). Where indicated, *p*-values have been adjusted to reflect one-tailed hypothesis tests. For effect sizes, we report Hedges' *g*. We standardized *g* by using the overall error term from the relevant linear model, and we corrected for bias using the procedure recommended by Borenstein *et al.* [42]. We report *R*^2^_change_ as the effect size for analyses testing whether the effect of religious priming is moderated by the extent to which subjects view God as a punishing, authoritarian figure.

**Table 4.** Descriptive statistics for DG transfers in Experiment 2.

priming method	religiosity	priming condition mean ± s.d. (*N*)		
		control	religious priming	total
explicit	non-religious	−0.030 ± 0.336 (52)	−0.082 ± 0.296 (39)	−0.052 ± 0.319 (91)
	religious	−0.009 ± 0.289 (96)	0.053 ± 0.290 (64)	0.016 ± 0.290 (160)
	total	−0.016 ± 0.306 (148)	0.002 ± 0.298 (103)	−0.009 ± 0.302 (251)
implicit	non-religious	−0.089 ± 0.321 (92)	−0.096 ± 0.328 (82)	−0.093 ± 0.324 (174)
	religious	−0.007 ± 0.317 (191)	−0.055 ± 0.324 (146)	−0.027 ± 0.320 (337)
	total	−0.033 ± 0.320 (283)	−0.070 ± 0.325 (228)	−0.050 ± 0.322 (511)
total	non-religious	−0.068 ± 0.327 (144)	−0.092 ± 0.317 (121)	−0.079 ± 0.322 (265)
	religious	−0.007 ± 0.307 (287)	−0.022 ± 0.317 (210)	−0.013 ± 0.311 (497)
	total	−0.028 ± 0.315 (431)	−0.047 ± 0.318 (331)	−0.036 ± 0.316 (762)

**Table 5.** Experiment 2 results summary: priming effects and interactions with religiosity.

prediction	model	mean difference	s.e.	*p-*values^a^	n1 prime	n2 control	*g*	s.e. of *g*	Bayes uniform	Bayes normal	Bayes 1/2 normal
1A: priming effect, all methods and subjects	GLM #1	−0.011	0.026	0.671	331	431	−0.04	0.07	0.08	0.05	0.13
1B: implicit priming effect, all subjects	GLM #2	−0.028	0.03	0.820	228	283	−0.09	0.09	0.07	0.05	0.11
1C: explicit priming effect, all subjects	GLM #3	0.005	0.04	0.452	103	148	0.02	0.13	0.21	0.13	0.31
2A: interaction with religiosity, all methods	GLM #1	0.037	0.050	0.237	331	431	—	—	1.05^b^	n.a.	n.a.
2A: interaction with religiosity, all methods	GLM #1	0.037	0.050	0.237	331	431	—	—	0.49^c^	n.a.	n.a.
2A: priming effect, all methods, religious subs	GLM #1	0.007	0.031	0.410	210	287	0.02	0.09	0.18	0.1	0.28
2A: priming effect, all methods, non-religious subs	GLM #1	−0.030	0.041	0.765	121	144	−0.10	0.12	0.1	0.07	0.16
2B: interaction with religiosity, implicit priming only	GLM #2	−0.04	0.059	0.750	228	283	—	—	0.00^d^	n.a.	n.a.
2B: interaction with religiosity, implicit priming only	GLM #2	−0.04	0.059	0.750	228	283	—	—	0.18^c^	n.a.	n.a.
2B: implicit priming effect, religious subs only	GLM #2	−0.048	0.035	0.912	146	191	−0.15	0.11	0.07	0.07	0.12
2B: implicit priming effect, non-religious subs only	GLM #2	−0.007	0.049	0.558	82	92	−0.02	0.15	0.16	0.1	0.25
2C: interaction with religiosity, explicit priming only	GLM #3	0.114	0.08	0.078	103	148	—	—	1.62^b^	n.a.	n.a.
2C: interaction with religiosity, explicit priming only	GLM #3	0.114	0.08	0.078	103	148	—	—	1.90^c^	n.a.	n.a.
2C: explicit priming effect, religious subs only	GLM #3	0.062	0.049	0.102	64	96	0.21	0.16	0.95	0.95	1.26
2C: explicit priming effect, non-religious subs only	GLM #3	−0.052	0.064	0.793	39	52	−0.17	0.21	0.17	0.12	0.25

^a^*p*-values are one-tailed.

^b^Bayes factor reflects original pre-registered analytical strategy.

^c^Bayes factor reflects revised analytical strategy.

^d^Bayes factor reflects original pre-registered analytical strategy, but the religious priming effect was in the direction counter to theory, rendering computation of the Bayes factor using pre-registered methods problematic.

**Table 6.** Experiment 2 results summary: interactions with view of God as authoritarian figure.

prediction	model	mean difference	s.e.	*p-*values^a^	Bayes factor uniform
‘A’ scale moderates priming effect, all methods	GLM #4	−0.001	0.025	0.518	1.07^b^
‘A’ scale moderates priming effect, all methods	GLM #4	−0.001	0.025	0.518	0.86^c^
‘A’ scale moderates priming effect, implicit priming only	GLM #5	0.004	0.030	0.452	0.00^d^
‘A’ scale moderates priming effect, implicit priming only	GLM #5	0.004	0.030	0.452	1.02^c^
‘A’ scale moderates priming effect, explicit priming only	GLM #6	−0.016	0.047	0.634	1.34^b^
‘A’ scale moderates priming effect, explicit priming only	GLM #6	−0.016	0.047	0.634	1.17^c^

^a^*p*-values are one-tailed.

^b^Bayes factor calculated from High/Low split.

^c^Bayes factor calculated from High/Low split; non-pre-registered analytical approach.

^d^Bayes factor calculated from High/Low split; the priming effect among subjects with highly authoritarian views of God was in the direction counter to theory, rendering computation of the Bayes factor using pre-registered methods problematic.

### 5.5. Did religious subjects transfer more money than did non-religious subjects?

Yes. As in Experiment 1, there were significant main effects of subject religiosity: among implicitly primed subjects, *F*_1,507_ = 4.27, *p* = 0.039 (two-tailed; GLM #2), and among subjects considered regardless of priming method, *F*_1,754_ = 7.49, *p* = 0.006 (two-tailed; GLM #1). For explicitly primed subjects, the effect of religiosity was marginally significant, *F*_1,247_ = 3.80, *p* = 0.052 (two-tailed; GLM #3). Across all subjects, religious subjects transferred an average of seven cents ($0.07) more than did non-religious subjects, *g* = 0.22, 95% CI [0.07, 0.37].

### 5.6. Prediction 1A: Was there a main effect of religious priming, such that the average Dictator Game transfer for all subjects receiving a religious prime (regardless of priming method) exceeded the average Dictator Game transfer for all subjects receiving a neutral prime?

No. Results from GLM #1 indicated that the DG transfers of religiously primed subjects (*M* = −$0.045) did not significantly differ from those of subjects who received a neutral prime (*M* = −$0.034), *t*_754_ = −0.443, s.e. = 0.026, *p* = 0.671 (one-tailed), *g* = −0.04, 95% CI [−0.18, 0.11]. Bayesian analyses of the non-significant finding supported the null hypothesis regardless of how we modelled the predictions of the priming hypothesis, with Bayes factors ranging from 0.05 (normal distribution) to 0.13 (half-normal distribution). These findings constitute evidence that religious priming did not increase DG transfers, considering subjects irrespective of their religiosity.

### 5.7. Prediction 1B: Was there a simple effect of implicit religious priming, such that the average Dictator Game transfer for all subjects receiving an implicit religious prime exceeded the average Dictator Game transfer for all subjects receiving an implicit neutral prime?

No. GLM #2 indicated that the DG transfers of subjects who received an implicit religious prime (*M* = −$0.075) did not differ significantly from those of subjects who received an implicit neutral prime (*M* = −$0.048), *t*_507_ = −0.915, s.e. = 0.030, *p* = 0.820 (one-tailed), *g* = −0.09, 95% CI [−0.26, 0.09]. Bayesian analyses of the non-significant finding supported the null hypothesis regardless of how we modelled the predictions of the priming hypothesis, with Bayes factors ranging from 0.05 (normal distribution) to 0.11 (half-normal distribution). These findings furnish evidence that implicit religious priming did not increase DG transfers, considering subjects irrespective of their religiosity.

### 5.8. Prediction 1C: Was there a simple effect of explicit religious priming, such that the average Dictator Game transfer for all subjects receiving an explicit religious prime exceeded the average Dictator Game transfer for all subjects receiving an explicit neutral prime?

No. GLM #3 indicated that the DG transfers of subjects who received an explicit religious prime (*M* = −$0.014) were not significantly greater than those of subjects who received an explicit neutral prime (*M* = −$0.019), *t*_247_ = 0.122, s.e. = 0.040, *p* = 0.452 (one-tailed), *g* = 0.02, 95% CI [−0.23, 0.27].

Bayesian analyses of the non-significant finding supported the null hypothesis regardless of how we modelled the predictions of the priming hypothesis, with Bayes factors ranging from 0.13 (normal distribution) to 0.31 (half-normal distribution). These findings constitute evidence that explicit religious priming did not increase DG transfers, considering subjects without regard to their religiosity. We note, however, that this conclusion does not hold if all subjects are included in the analyses. If the full sample (without exclusions) is analysed, the data appear to be insensitive to a possible effect of explicit priming, based on Bayes factors generated using both the uniform distribution (0.38) and the half-normal distribution (0.57) to model the predictions of the priming hypothesis. See electronic supplementary material, table S5.

### 5.9. Prediction 2A: Was there a significant two-way interaction such that the main effect of religious priming (regardless of priming method) was greater for religious subjects than for non-religious subjects, and the effect on non-religious subjects did not differ statistically from zero?

No. Results from GLM #1 indicated that the interaction of priming condition with subject religiosity was not significant, *t*_754_ = 0.717, s.e. = 0.051, *p* = 0.237 (one-tailed). There was no significant effect of religious priming, either for religious subjects (*t*_754_ = 0.23, s.e. = 0.031, *p* = 0.410 (one-tailed), *g* = 0.02, 95% CI [−0.16, 0.20]) or for non-religious subjects (*t*_754_ = −0.73, s.e. = 0.041, *p* = 0.765 (one-tailed), *g* = −0.10, 95% CI [−0.33, 0.15]). Regarding this interaction, Bayesian analyses conducted using our revised methods (see Data analyses) were unable to distinguish the null hypothesis from the priming hypothesis, with a Bayes factor equal to 0.49, indicating that the data were insensitive to the interaction effect.

When we examined the simple effects of religious priming, Bayesian analyses provided evidence that religious priming did *not* affect the DG transfers of specifically *religious* subjects, with Bayes factors ranging from 0.10 (normal distribution) to 0.28 (half-normal distribution). Bayesian analyses likewise furnished reliable evidence that religious priming had no effect on specifically *non-religious* subjects, with Bayes factors ranging from 0.07 (normal distribution) to 0.16 (half-normal distribution).

### 5.10. Prediction 2B: Among implicitly primed subjects only, was there a significant interaction, such that the simple effect of implicit religious priming was positive and greater for religious subjects than for non-religious subjects, and that the effect on non-religious subjects did not differ statistically from zero?

No. The interaction of priming condition with subject religiosity was not significant in GLM #2, *t*_507_ = −0.676, s.e. = 0.059, *p* = 0.750. There was no significant effect of implicit religious priming, either for religious subjects (*t*_507_ = −1.37, s.e. = 0.035, *p* = 0.912 (one-tailed), *g* = −0.15, 95% CI [−0.36, 0.07]) or for non-religious subjects (*t*_507_ = −0.14, s.e. = 0.049, *p* = 0.558 (one-tailed), *g* = −0.02, 95% CI [−0.32, 0.28]).

Our revised analytical methods (see Data analyses) resulted in a Bayes factor of 0.18, supporting the null hypothesis of no interaction. When we considered the simple effects of implicit religious priming, Bayesian analyses provided evidence that implicit religious priming did *not* affect the DG transfers of specifically *religious* subjects, with Bayes factors ranging from 0.07 (normal distribution) to 0.12 (half-normal distribution). Similarly, Bayesian analyses furnished reliable evidence that implicit religious priming had no effect on specifically *non-religious* subjects, with Bayes factors ranging from 0.10 (normal distribution) to 0.25 (half-normal distribution). Altogether, these analyses indicate that the effect of implicit religious priming did not differ based on religiosity of the subjects, *and* that the priming effect was zero rather than positive for both non-religious and religious subjects alike.

### 5.11. Prediction 2C: Among explicitly primed subjects only, was there a significant interaction, such that the simple effect of explicit religious priming was positive and greater for religious subjects than for non-religious subjects, and that the effect on non-religious subjects did not differ statistically from zero?

No. In GLM #3, the interaction of priming condition with subject religiosity was marginally significant using a one-tailed test, *t*_247_ = 1.43, s.e. = 0.080, *p* = 0.078 (one-tailed). There was no significant effect of explicit religious priming, either for religious subjects (*t*_247_ = 1.27, s.e. = 0.049, *p* = 0.102 (one-tailed), *g* = 0.21, 95% CI [−0.11, 0.52]) or for non-religious subjects (*t*_247_ = −0.81, s.e. = 0.064, *p* = 0.793 (one-tailed), *g* = −0.17, 95% CI [−0.59, 0.24]).

For the interaction, Bayesian analyses conducted using our revised methods (see Data analyses) were unable to distinguish the null hypothesis from the priming hypothesis, yielding a Bayes factor = 1.90. An analysis of the simple effects of explicit priming did not exclude the possibility that explicit priming of specifically *religious* subjects increases DG transfers, with Bayes factors ranging from 0.95 (uniform distribution) to 1.26 (half-normal distribution). However, Bayesian analyses of the simple effect of priming on specifically *non-religious* subjects did argue against the priming hypothesis, with Bayes factors ranging from 0.12 (normal distribution) to 0.25 (half-normal distribution). We note that this conclusion is perhaps slightly qualified if the full sample (without exclusions) is analysed. When we modelled the priming hypothesis using the half-normal distribution and the entire sample, the resulting Bayes factor (0.36) suggested that the data may be insensitive (see electronic supplementary material, table S5).

Overall, the Experiment 2 data tend to argue against an effect of explicit priming on non-religious subjects, but do not exclude an effect of explicit religious priming on religious subjects, or different effects of explicit religious priming for religious versus non-religious subjects.

### 5.12. Prediction 3: Did the effect of implicit religious priming remain significant even after removing from the analysis any subjects who reported conscious awareness of religious words during the suspicion probe?

Because there were no significant effects of implicit religious priming, we did not perform this analysis.

### 5.13. Prediction 4: Were the effects of religious priming moderated by the extent to which subjects viewed God as authoritarian?

There were 341 Experiment 2 subjects who identified as Christian after exclusions for suspicion, essay length and insufficient attention to the task. This subsample formed the basis of the moderation analyses that follow. To test for moderation of the religious priming effect, we regressed DG transfers on priming condition, subject's authoritarian view of God and the interaction of the two.

*GLM #4: Results for all Christian subjects, regardless of priming method.* Using all 341 Christian subjects, we found no significant interaction between authoritarian views of God and priming condition, *t*_336_ = −0.045, s.e. = 0.025, *p* = 0.518 (one-tailed), *R*^2^_change_ = 0.000.

To interpret this non-significant result using Bayesian analyses, we split the sample of Christian subjects into two groups based on authoritarian views of God, as described previously. After we did so, 121 subjects remained, with cell sizes ranging from 39 (for neutrally primed subjects with low authoritarian views of God) to 25 (for religiously primed subjects with low authoritarian views of God, and for neutrally primed subjects with high authoritarian views of God). There was no significant interaction between authoritarian views of God and priming condition, *t*_117_ = 0.592, s.e. = 0.119, *p* = 0.277 (one-tailed). Bayesian analyses of this non-significant interaction (using our revised analytical strategy—see Data analyses) returned a Bayes factor of 0.86, indicating that the data were insensitive rather than being supportive of the null.

*GLM #5: Results for implicitly primed Christian subjects.* We then restricted the sample to implicitly primed Christians (*N* = 234) before testing whether authoritarian views of God moderated the effect of religious priming on DG transfers. The interaction was not significant, *t*_229_ = 0.120, s.e. = 0.030, *p* = 0.452 (one-tailed), *R*^2^_change_ = 0.000.

To perform Bayesian analyses of this non-significant result, we again split the sample of Christian subjects into two groups based on authoritarian views of God. After we did so, 86 subjects remained, with individual cell sizes ranging from 17 (for religiously primed subjects with low authoritarian views of God) to 25 (for neutrally primed subjects with low authoritarian views of God). Using this subsample to regress DG transfers on priming condition, the grouping variable that captured authoritarian views of God (High versus Low), and the interaction of the two, we found that the interaction between authoritarian views of God and priming condition was not significant, *t*_82_ = 0.572, s.e. = 0.142, *p* = 0.284 (one-tailed). Using our revised analytical strategy (see Data analyses), we obtained a Bayes factor of 1.02, indicating that the data provided no evidence against the null hypothesis.

*GLM #6: Results for explicitly primed Christian subjects.* Finally, we tested for moderation of the priming effect after restricting the sample to explicitly primed Christian subjects (*N* = 107). The regression of priming condition, authoritarian views of God and the interaction of the two yielded a non-significant interaction, *t*_103_ = −0.342, s.e. = 0.047, *p* = 0.634 (one-tailed), *R*^2^_change_ = 0.001.

To interpret this null result using our pre-registered Bayesian analyses, we split the sample of Christian subjects into two groups, based on whether they viewed God as high or low in authoritarianism. This resulted in a subsample of 35 subjects, with individual cell sizes ranging from 4 (for neutrally primed subjects with highly authoritarian views of God) to 14 (for neutrally primed subjects with low authoritarian views of God). When we regressed DG transfers on priming condition, the grouping variable that captured authoritarian views of God (High versus Low), and the interaction of the two, the interaction was not significant, *t*_31_ = 0.891, s.e. = 0.212, *p* = 0.190 (one-tailed). Bayesian analyses using our revised analytical strategy (see Data analyses) indicated that the data were insensitive rather than supportive of the null (Bayes factor = 1.17).

Considering all of the moderation analyses for Experiment 2 together, we thus find that the data do not rule out the possibility that the effect of religious priming among Christian subjects varies as a function of how authoritarian the subject understands God to be.

### 5.14. Discussion

To test whether religious priming increases DG transfers, Experiment 2 used a modified DG that gave dictators the option to take as well as to give money. Apart from this innovation—which was introduced to encourage high baseline levels of selfish behaviour—Experiment 2 closely paralleled Experiment 1, making use of the same implicit and explicit primes. We again obtained consistently non-significant results and used Bayesian analyses to assess whether the data were insensitive or actively supported the null hypothesis.

When we considered the combined results—irrespective of priming method or subjects' religiosity—we found substantial evidence favouring the null hypothesis that religious priming does not increase DG transfers. In contrast to what we found for Experiment 1, however, the conclusions suggested by our Bayesian analyses did not vary according to the way we chose to model the predicted distribution of the priming effect, and the combined data were anywhere from 7.5 to 20 times more likely under the null than under the priming hypothesis. When we examined implicit and explicit priming separately, we found evidence that neither method increased DG transfers among subjects considered without regard to their religiosity. Evidence against an implicit priming effect was very strong: the data were 9–20 times more likely under the null hypothesis (for explicit priming, the data were 3–7.5 times more likely under the null).

Perhaps, the effects of religious priming were to be found only among religious subjects? For implicit priming, the Experiment 2 data indicated that this was not the case, providing substantial evidence *against* an implicit priming effect in religious subjects and in non-religious subjects, along with evidence that the effect did not vary by religiosity. For explicit priming, the picture may be more nuanced. Experiment 2 argued against an effect of explicit priming among non-religious subjects, but it offered no evidence against the proposition that explicit priming increases DG transfers among religious subjects, with a small effect size (*g* = 0.21).

Like Experiment 1, Experiment 2 had the secondary goal of testing whether the effect of religious priming (among Christian subjects only) depended upon the extent to which subjects conceptualized God as a punishing, authoritarian figure. Our results, though non-significant, failed to provide any meaningful evidence in favour of the null hypothesis. Experiment 2 thus does not argue against the possibility that the effect of religious priming among Christian subjects may vary as a function of how punishing or authoritarian they understand God to be.

## 6. Study 3

We updated Gomes and McCullough's meta-analysis [28] with the results of our implicit priming data, using a random-effects meta-analysis, PET-PEESE estimation, and the trim and fill procedure employed by Shariff *et al*. [12]. These meta-analyses included the data from Gomes & McCullough [28], the data collected in the two experiments performed here, and new experiments by other investigators of which we became aware. Finally, we conducted a random-effects meta-analysis on available *pre-registered* replications (including those presented herein) that used implicit priming, thus obviating the need for bias-corrected meta-analytical estimates.

Our search for new studies generally followed the approach of Shariff *et al*. [12], but was restricted to experiments using the DG as the outcome measure, which greatly circumscribed the number of relevant studies. First, we searched Google Scholar, PsycINFO and Web of Knowledge for studies that were published in 2014 or later and that contained the keywords *dictator*, prim*, god and relig*. Second, we used Google Scholar to determine which studies published in 2014 or later cited any of [12,23,28]. Third, we searched the website of the Society of Personality and Social Psychology for relevant presentations at any of their annual conferences since 2014, using the key word relig*. Finally, on 18 January 2018, we sent out a call for religious priming experiments on the SPSP listserv. Our efforts revealed one new experiment that used implicit religious priming in a Japanese sample [43].

All meta-analyses were conducted using the metafor package in R v. 3.3.2 [44] and reflect the Knapp and Hartung adjustment for small sample sizes ([45,46]; see also [47,48]). Effect sizes were Hedges' *g*. For studies that appeared in Gomes & McCullough [28], we determined Hedges’ *g* and the associated variance from their table 3. For Miyatake & Higuchi [43], we calculated Hedges' *g* and the associated variance from the reported *t-*value and sample sizes. We then added the results of our own Experiment 1 and Experiment 2 for implicitly primed subjects only.

### 6.1. Results

**Figure 1.**
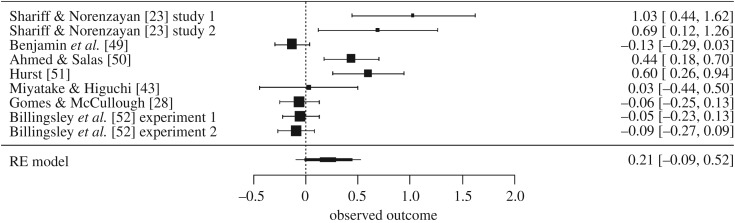
Random-effects meta-analysis of all implicit religious priming studies with DG transfers as the outcome.

Figure 1 presents the results of the meta-analysis of all nine studies, which includes both pre-registered and non-pre-registered experiments and considers participants irrespective of religiosity or other moderating variables. Without adjusting for possible publication bias, the overall weighted effect size did not differ statistically from zero, *g* = 0.21, s.e. = 0.13, *p* = 0.146 95% CI [−0.09, 0.52]. We found evidence that significant variation in effect size is attributable to between-study differences rather than sampling error, *Q* = 43.43, d.f. = 8, *p* < 0.001, *I*^2^ = 88.5%. Egger's test for funnel plot asymmetry [53] revealed evidence of publication bias, *t* = 3.72, d.f. = 7, *p* = 0.008. Results of the trim and fill procedure, which tends to undercorrect for publication bias [54], suggested an estimated weighted effect size of *g* = 0.09, s.e. = 0.14, *p* = 0.516, 95% CI [−0.19, 0.38]. The PET-PEESE procedure, which tends to overcorrect for publication bias [54], estimates the effect size *g* that would be produced in an idealized experiment with a standard error of zero—and thus of infinite precision—by regressing effect size on standard error and interpreting the intercept (*b*_0_) as the expected effect size under those idealized conditions. In doing so with our sample, we found that *b*_0_ = −0.45, s.e. = 0.14, *p* = 0.016, 95% CI [−0.78, −0.11]. When the resulting intercept is significantly different from zero, as is the case here, Stanley *et al*. [55] suggest that a more accurate estimate of effect size is obtained from a regression of effect size on variance. Performing this regression, we found that *b*_0_ = −0.13, s.e. = 0.08, *p* = 0.165, 95% CI [−0.33, 0.07]. PET-PEESE estimation thus suggests that the effect size of implicit religious priming on DG transfers does not differ statistically from zero.

**Figure 2.**
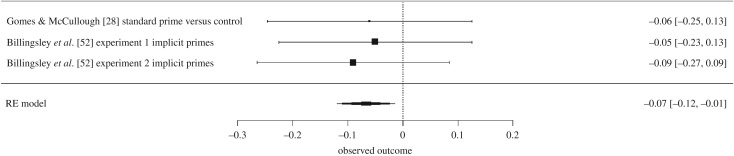
Random-effects meta-analysis of all pre-registered implicit religious priming studies with DG transfers as the outcome.

As an alternative approach to addressing publication bias, we performed a second meta-analysis using only pre-registered experiments. Here, as figure 2 shows, the overall weighted effect size was small and just barely statistically different from zero, albeit in the opposite direction predicted by the religious prosociality hypothesis, *g* = −0.07, s.e. = 0.01, *p* = 0.032, 95% CI [−0.12, −0.01]. We found no variation in effect sizes that was attributable to between-study differences, *Q* = 0.11, d.f. = 2, *p* = 0.948, *I*^2^ = 0.00%.

## 7. General discussion

Prominent theoretical approaches to understanding religion hold that widespread forms of religious belief promote prosocial behaviour. Because the claim is causal, proponents of these theories have sought key empirical support from a growing body of experiments based on the use of religious priming methods. Consistent with these theories, a recent meta-analysis provided evidence for a small but reliable effect of religious priming on prosociality [12], although other meta-analytical findings [19] and a pre-registered replication [28] have led to more circumspect conclusions.

In two pre-registered experiments, supplemented by a small-scale meta-analysis, we sought to shed light on the lingering issues concerning the religious priming evidence. To do so, we focused on a specific empirical question: ‘Does religious priming—implicit, explicit, or both—increase DG transfers?’

Overall, the pattern of results from these two experiments pointed to one clear finding. Namely, both experiments provided evidence that *implicit* religious priming does not increase monetary transfers in the DG. In both experiments, subjects who were implicitly primed with religious concepts did not transfer more money than did subjects implicitly primed with neutral concepts. Indeed, across both experiments, visual inspection of the raw means would suggest that, if anything, subjects who received an implicit religious prime might have transferred *less* money, not more, relative to subjects who received a neutral implicit prime. Most crucially, however, we conducted Bayesian analyses, which permitted us to interpret our non-significant results. For both experiments, these analyses substantially favoured the hypothesis that implicit religious priming has no effect on DG transfers. This conclusion held when we analysed the data without excluding any participants.

The possibility that implicit priming has no effect on DG transfers may appear to stand in some tension with major conclusions highlighted in Shariff *et al*.'s meta-analysis [12]. For instance, Shariff *et al*. [12] analysed 92 studies involving a wide range of outcomes (including dependent variables not related to prosociality) and found that implicit religious priming had a reliable, small-to-medium effect (*g* = 0.39) that did not differ markedly from the mean effect size for explicit religious priming (*g* = 0.42). When Shariff *et al*. restricted their meta-analysis to studies involving prosociality, they reported that religious priming overall—but not necessarily implicit religious priming specifically—still had a significant effect, even adjusting for publication bias (*g* = 0.18) [12]. It is not clear from Shariff *et al*.'s meta-analysis, however, whether there was a significant effect for the 12 (out of 25) studies of prosocial behaviour that used implicit priming methods, or whether there was a significant effect for religious priming studies involving specifically the DG, irrespective of priming method. Gomes & McCullough [28] partially addressed these gaps in their small-scale meta-analysis of the six available implicit religious priming studies involving the DG. Gomes and McCullough found no statistically significant effect of implicit religious priming on DG transfers, although the 95% confidence interval was wide and contained many positive values. Here, we updated Gomes & McCullough [28] with a new meta-analysis that included results from nine implicit priming experiments. After adjusting for publication bias, we found that the estimated effect of implicit priming on DG transfers was not significantly different from zero. When we meta-analysed only the three pre-registered experiments, the estimated effect size was negative, with a 95% confidence interval that fell just short of zero.

### 7.1. Boundary conditions?

Although Experiments 1 and 2 provided evidence that implicit priming does not increase DG transfers in general, proponents of the priming hypothesis might attribute the null results to one or more boundary conditions hypothesized to circumscribe the effect [29]. For instance, the religious priming effect might be absent or highly attenuated among individuals who are already motivated to behave prosocially [29]. Several facts, however, speak against this possibility in the present experiments. First, subjects in the control condition of Experiment 1 transferred only 28.4% of their endowment on average, well within the 14–33% range that Shariff & Norenzayan [29] speculated as necessary for producing a religious priming effect on DG transfers by reducing selfish motivation. Second, the DG transfers we observed in our Experiment 1 control condition are also in accord with the results of a pre-registered online pilot study that we conducted in September 2015, using 200 unprimed subjects from Amazon's Mechanical Turk (see pre-registration at https://osf.io/2ghv6/). Following the same DG instructions as in Experiment 1 here, subjects transferred 24.1% of their endowments on average, which militates against the possibility that the baseline prosocial motivation of the Mechanical Turk population is too high for religious priming to exert an effect [29]. Third, there are the results from the control conditions of Experiment 2: average transfers across all neutral conditions in Experiment 2 were negative in sign, indicating that the average subject took money instead of giving money. This suggests that Experiment 2 subjects were not motivated to be ‘hyperfair’. Altogether, there is little reason to suspect that our null results in Experiments 1 and 2 are due to unusually high levels of pre-existing prosocial motivation.

Another boundary condition that could be invoked to explain why we found no overall effect of implicit priming is that the effect obtains only with religious subjects. Indeed, Shariff *et al*. [12] reported that the average effect of religious priming on prosocial behaviour (across 17 studies that used explicit as well as implicit priming methods) was statistically indistinguishable from zero among non-religious subjects, after adjusting for publication bias. Experiments 1 and 2, however, showed that implicit priming does not increase DG transfers among religious subjects (or non-religious ones).

It is also unlikely that our evidence against the efficacy of implicit religious priming on DG transfers reflects characteristics peculiar to the Mechanical Turk population. Shariff *et al*.'s meta-analysis of 92 religious priming studies included numerous experiments drawing upon either Mechanical Turk (13 studies) or other online platforms for data collection (12 studies) [12]. Shariff *et al*. [12] reported no differences in the effect of religious priming as a function of experimental setting, whether that setting was the laboratory, the field, Mechanical Turk or another online platform. Indeed, Shariff *et al*.'s meta-analysis provided little reason for concern that religious priming has significantly less potent effects on Mechanical Turk or other online platforms, relative to field or laboratory settings.

### 7.2. Implicit versus explicit religious priming

Experiments 1 and 2 converged in providing evidence against an effect of implicit religious priming. In important respects, they also converged regarding the possible effects of *explicit* religious priming. Breaking down the samples by religiosity helps to clarify the nature of this convergence. If we consider only religious subjects, Experiments 1 and 2 produced nearly identical estimates of the effect of explicit religious priming, and with almost identical confidence intervals (*g* = 0.20 and 0.21, respectively). The effect size for each experiment, taken individually, did not statistically differ from zero. When combined, however, the two effect size estimates provide evidence of a small but reliable effect of explicit priming on religious subjects: a random-effects meta-analysis suggests that the composite effect size for explicit priming of religious subjects is *g* = 0.21, s.e. = 0.01, *p* = 0.016, 95% CI [0.14, 0.27], which is reasonably consistent with conclusions from Shariff *et al*. [12], who reported a meta-analytical effect size of religious priming upon ‘religious/high religiosity’ participants (regardless of priming method) of *g* = 0.28, after correcting for publication bias. Though it is worthwhile to note that the effect size estimate we obtained here was so small that one would need a sample of 620 religious subjects to achieve 80% statistical power for detecting it (using the methods of our experiments), the effect appears to be reliably larger than zero.

Although our two experiments converged with regard to an effect of explicit priming on religious participants, the two experiments diverged concerning the possibility of an explicit priming effect among *non-religious* individuals. Experiment 2 offered substantive evidence against the possibility; Experiment 1 did not. Although the results of Experiment 1 were inconclusive with regard to an explicit priming effect among non-religious subjects, we suggest that the results of Experiment 2 together with the findings from Shariff *et al*.'s meta-analysis [12] provide sufficient reason to suspect that the effect of explicit priming on non-religious individuals is either absent or very weak—perhaps to the point of practical insignificance.

### 7.3. An effect of religiosity

Our two experiments thus argue against an effect of implicit religious priming upon DG transfers, collectively provide evidence for a small effect of explicit priming upon religious individuals and furnish new grounds to suspect that explicit priming has little or no effect on the transfers of non-religious individuals. We also note that in both experiments religious individuals transferred more money on average than did non-religious individuals—8.6 cents more in Experiment 1, and 7.0 cents more in Experiment 2. This effect of religiosity emerged regardless of priming condition and appears to add to the body of behavioural research suggesting a positive association of religiosity with prosociality [1,2]. It is interesting that a persistent effect of religiosity emerged even though the anonymous recipients of these transfers had no previous relationships with the subjects and were unable to convey any information about whether they actually had a need for some of the dictator's money. As closeness and need are two of the major social–psychological cues influencing people's regard for others’ welfare, it is not entirely clear that religious people's larger transfers in the two experiments reported here reflect a heightened regard for the welfare of strangers *per se*. Some researchers have suggested instead that DG transfers may largely reflect a desire to avoid negative social evaluation [56–58], which raises the possibility that results such as those we obtained here reflect instead a greater tendency for religious people to share with others out of a heightened aversion to negative social evaluation for seeming stingy.

### 7.4. Supernatural punishment

Unfortunately, our experiments shed little light on the possibility that the effect of religious priming upon DG transfers is stronger to the extent that subjects view God as a punishing, authoritarian figure. In testing whether the effect of religious priming is moderated by how authoritarian subjects understand God to be, we consistently obtained non-significant results, but Bayesian analyses showed that the data offer no warrant for favouring the null hypothesis. The data were insensitive regardless of whether we examined implicit and explicit priming separately, and regardless of whether we broke down results by subject religiosity. Our ability to address this research question effectively was hampered by the small sample sizes available for the Bayesian analyses. Future research involving larger samples will be needed to investigate this question further.

## 8. Conclusion

We conclude on a practical note. If, as our research suggests, implicit religious priming has little or no reliable effect on DG transfers, future researchers might consider turning their attention to explicit and contextual primes. Future pre-registered experiments with explicit religious primes might help to clarify the reliability and magnitude of the hypothesized priming effects, illuminate the extent to which any such effects may be artefacts of experimental demand and test hypotheses concerning the specific psychological processes by which religious cognition might increase prosocial behaviour. By attending to such methodological priorities, researchers can help to provide the experimental psychology of religion with a sound empirical basis from which to initiate and to evaluate theory.

## Supplementary Material

Supplemental Information

## Data accessibility

Data are publicly available through the *Open Science* Framework at the following link: https://osf.io/6nqwt/.

## Appendix A. Text of priming conditions

.

.

.

## Appendix B. Comparison of methods and instructions used in Shariff & Norenzayan [23] and in our Experiment 1 (implicit primes)

.

.

.

.

.

.

## Appendix C. Experiment 2 DG Instructions

.
