# Supplementary material for: Implicit and explicit influences of religious cognition on Dictator Game transfers
Source: R Soc Open Sci. 2018 Aug 22;5(8):170238. doi: 10.1098/rsos.170238 (PMC6124061; doi:10.1098/rsos.170238)
Supplement: Supplemental Information [file rsos170238supp1.docx]

**Supplemental Online Information for**

**Implicit and Explicit Influences of Religious Cognition Upon Dictator Game Transfers**

**by Joseph Billingsley, Cristina M. Gomes, and Michael E. McCullough**

*Subject Recruitment and Exclusion.* For each experiment, we intended to recruit 776 usable subjects from Amazon’s Mechanical Turk population for a 2 (Priming Condition: Religious vs. Control) x 2 (Priming Method: Explicit vs. Implicit) x 2 (Religiosity: Religious vs. Non-religious) between-subjects design. Our pre-registration specified that we would attempt to arrive at 194 usable subjects (after exclusions) in each of four major groups: (1) subjects given the explicit religious prime; (2) subjects given the explicit control prime; (3) subjects given the implicit religious prime; and (4) subjects given the implicit control prime. We also pre-registered an iterative recruitment procedure that provided for recruitment of additional subjects after exclusions based on pre-specified criteria. This process could have resulted in major budget over-runs, so we pre-registered that we would cease running subjects at 932 total subjects per experiment, even if the target of 194 usable subjects per cell was not reached.

Subjects were required to have a minimum 90% approval rate on MTurk for previously performed tasks and to reside in the United States. For each subject, we advertised a task offering a modest payment for completing a 15-20 minute experiment involving one or more decision-making tasks, questionnaires, and/or writing tasks. Subjects were also informed that they might receive additional money depending on choices made during the set of decision-making tasks.

Data were collected for both experiments simultaneously. During data collection, we encountered substantially higher dropout rates in the explicit versus implicit conditions. Suspecting that this was due to some subjects not wishing to write an essay, we elected not to break randomization, even though it meant that we would be well short of the targeted 194 subjects per cell for the explicit priming conditions when we reached our first pre-registered recruitment goal of 776 usable subjects per experiment. We therefore recruited additional subjects in order to obtain at least 932 subjects for each experiment—the maximum that our resources would allow, as specified in the pre-registration.

1,909 subjects finished the experiments—949 for Experiment 1 and 960 for Experiment 2. For Experiment 1, the sample included 595 implicitly primed subjects and 353 explicitly primed subjects. For Experiment 2, the sample included 608 implicitly primed subjects and 352 explicitly primed subjects. The higher dropout rate in the explicit priming conditions thus resulted in more implicitly primed subjects per cell than expected (approximately 300 per cell before exclusions, rather than 194), but fewer explicitly primed subjects per cell than expected (approximately 176 per cell before exclusions, rather than 194).

Subjects were excluded in accordance with pre-registered criteria. Responses to the first, second, and fifth items from a suspicion probe (see Appendix B) were reviewed by research assistants who were blind to other subject data. Subjects whose responses to any of these three items linked religion to any form of prosociality (e.g. generosity, altruism, charity, giving to others, being fair, etc.) were excluded from all analyses. Research assistants coded 82 Experiment 1 subjects (8.6%) and 72 Experiment 2 subjects (7.5%) as suspicious on these grounds. Moreover, subjects in implicit priming conditions who did not correctly complete all ten scrambled sentences were excluded from analysis. Because the scrambled sentences in many cases permitted multiple correct solutions, we adopted a coding system with four possible responses to each item: “Clearly correct,” “Arguably correct,” “Incorrect,” and “Missing.” Subjects with one or more scrambled sentences coded “Incorrect” or “Missing” were excluded from the main analyses. Finally, research assistants determined the word count of each essay by copying the text into Microsoft Word and entering the displayed word count into the data file. We then excluded any essay that was less than 50 words in length. By following this we excluded 47 (5%) in Experiment 1 and 39 subjects (4.1%) in Experiment 2. Altogether, 218 Experiment 1 subjects (23%) and 192 Experiment 2 subjects (20%) were excluded.

**Table S1. Descriptive Statistics for Dictator Game Transfers in Experiment 1, No Subjects Excluded**

|  |  | **Priming Condition**  Mean +/- SD (N) | | |
| --- | --- | --- | --- | --- |
| Priming Method | Religiosity | Control | Religious Priming | Total |
| Explicit | Non-religious | .235 +/- .280  (55) | .283 +/- .288  (63) | .260 +/- .284  (118) |
|  | Religious | .314 +/- .273  (114) | .369 +/- .333  (121) | .342 +/- .306  (235) |
|  | Total | .288 +/- .277  (169) | .339 +/- .320  (184) | .315 +/- .301  (353) |
| Implicit | Non-religious | .246 +/- .279  (102) | .220 +/- .277  (104) | .233 +/- .278  (206) |
|  | Religious | .301 +/- .269  (183) | .289 +/- .294  (203) | .295 +/- .282  (386) |
|  | Total | .281 +/- .273  (285) | .266 +/- .290  (307) | .273 +/- .282  (595) |
| Total | Non-religious | .242 +/- .279  (157) | .244 +/- .282  (167) | .243 +/- .280  (324) |
|  | Religious | .306 +/- .270  (297) | .319 +/- .311  (324) | .313 +/- .292  (621) |
|  | Total | .284 +/- .274  (454) | .293 +/- .303  (491) | .289 +/- .290  (945) |

**Table S2. Experiment 1 Results Summary, No Subjects Excluded: Priming Effects and Interactions with Religiosity**

| **Prediction** | **Model** | **Mean**  **Diff** | **SE** | **p^*^** | **n1 Prime** | **n2**  **Ctrl** | ***g*** | **se of *g*** | **Bayes**  **Factor**  **Uniform** | **Bayes**  **Factor**  **Normal** | **Bayes**  **Factor**  **½ Normal** |
| --- | --- | --- | --- | --- | --- | --- | --- | --- | --- | --- | --- |
| 1A: Priming effect, all methods | GLM#1 | .016 | .020 | .216 | 491 | 454 | 0.06 | 0.07 | .24 | .12 | .37 |
| 1B: Implicit priming effect, all subjects | GLM#2 | -.019 | .024 | .785 | 307 | 285 | -0.07 | 0.08 | .08 | .05 | .12 |
| 1C: Explicit priming effect, all subjects | GLM#3 | .051 | .034 | .065 | 184 | 169 | 0.17 | 0.11 | 1.08 | 1.02 | 1.49 |
| 2A: Interaction with religiosity,  all methods | GLM#1 | .01 | .04 | .404 | 491 | 454 | - | - | 1.02** | N/A | N/A |
| 2A: Interaction with religiosity,  all methods | GLM#1 | .01 | .04 | .404 | 491 | 454 | - | - | .33*** | N/A | N/A |
| 2A: Priming effect, all methods,  Religious subs only | GLM#1 | .021 | .024 | .188 | 324 | 297 | 0.07 | 0.08 | .37 | .25 | .56 |
| 2A: Priming effect, all methods,  Non-religious subs only | GLM#1 | .011 | .033 | .370 | 167 | 157 | 0.04 | 0.11 | .21 | .13 | .33 |
| 2B: Interaction with religiosity,  Implicit priming only | GLM#2 | .014 | .05 | .432 | 307 | 285 | - | - | 0.00† | N/A | N/A |
| 2B: Interaction with religiosity,  Implicit priming only | GLM#2 | .014 | .05 | .432 | 307 | 285 | - | - | .40*** | N/A | N/A |
| 2B: Implicit priming effect,  Religious subs only | GLM#2 | -.012 | .029 | .668 | 203 | 183 | -0.04 | 0.10 | .13 | .08 | .21 |
| 2B: Implicit priming effect,  Non-religious subs only | GLM#2 | -.026 | .039 | .746 | 104 | 102 | -0.09 | 0.14 | .12 | .08 | .19 |
| 2C: Interaction with religiosity,  Explicit priming only | GLM#3 | .0066 | .067 | .462 | 184 | 169 | - | - | .93** | N/A | N/A |
| 2C: Interaction with religiosity,  Explicit priming only | GLM#3 | .0066 | .067 | .462 | 184 | 169 | - | - | .65*** | N/A | N/A |
| 2C: Explicit priming effect,  Religious subs only | GLM#3 | .055 | .039 | .081 | 121 | 114 | 0.18 | 0.13 | 1.31 | 1.43 | 1.63 |
| 2C: Explicit priming effect,  Non-religious subs only | GLM#3 | .048 | .055 | .192 | 63 | 55 | 0.16 | 0.18 | .62 | .58 | .84 |

*Note: *p*-values are one-tailed

**Bayes factor reflects original pre-registered analytic strategy

***Bayes factor reflects revised analytic strategy

†Bayes factor reflects original pre-registered analytic strategy, but the religious priming effect was in the direction counter to theory, rendering computation of the Bayes factor using pre-registered methods problematic.

**Table S3, Experiment 1 Results Summary, No Subjects Excluded: Interactions with View of God as Authoritarian Figure**

| **Prediction** | **Model** | **Mean**  **Diff** | **SE** | ***p**** | **Bayes**  **Factor**  **Uniform** |
| --- | --- | --- | --- | --- | --- |
| “A” Scale moderates priming effect, all methods | GLM#1 | .011 | .022 | .313 | 0.00† |
| “A” Scale moderates priming effect, all methods | GLM#1 | .011 | .022 | .313 | .75*** |
| “A” Scale moderates priming effect,  Implicit priming only | GLM#2 | .026 | .028 | .181 | 1.04** |
| “A” Scale moderates priming effect,  Implicit priming only | GLM#2 | .026 | .028 | .181 | 2.57*** |
| “A” Scale moderates priming effect,  Explicit priming only | GLM#3 | .000 | .034 | .500 | .99** |
| “A” Scale moderates priming effect,  Explicit priming only | GLM#3 | .000 | .034 | .500 | .58*** |

Note: *p*-values are one-tailed

**Bayes Factor calculated from High/Low split

***Bayes Factor calculated from High/Low split; non-preregistered analytic approach

†Bayes Factor calculated from High/Low split; the priming effect among subjects with highly authoritarian views of God was in the direction counter to theory, rendering computation of the Bayes factor using pre-registered methods problematic.

**Table S4. Dictator Game Transfers, Experiment 2, No Subjects Excluded**

|  |  | **Priming Condition**  Mean +/- SD (N) | | |
| --- | --- | --- | --- | --- |
| Priming Method | Religiosity | Control | Religious Priming | Total |
| Explicit | Non-religious | -.037 +/- .343  (56) | -.036 +/- .307  (70) | -.306 +/- .323  (126) |
|  | Religious | -.018 +/- .298  (111) | .043 +/- .289  (115) | .013 +/- .294  (226) |
|  | Total | -.024 +/- .313  (167) | .013 +/- .297  (185) | -.005 +/- .305  (352) |
| Implicit | Non-religious | -.091 +/- .320  (103) | -.088 +/- .327  (102) | -.090 +/- .322  (205) |
|  | Religious | .000 +/- .319  (209) | -.041 +/- .323  (187) | -.019 +/- .321  (396) |
|  | Total | -.030 +/- .321  (312) | -.058 +/- .325  (289) | -.043 +/- .323  (601) |
| Total | Non-religious | -.072 +/- .328  (159) | -.067+/- .319  (172) | -.069 +/- .323  (331) |
|  | Religious | -.006 +/- .311  (320) | -.009 +/- .313  (302) | -.008 +/- .312  (622) |
|  | Total | -.028 +/- .318  (479) | -.030 +/- .316  (474) | -.029 +/- .317  (953) |

**Table S5. Experiment 2 Results Summary, No Subjects Excluded: Priming Effects and Interactions with Religiosity**

| **Prediction** | **Model** | **Mean**  **Diff** | **SE** | ***p*^*^** | **n1 Prime** | **n2**  **Ctrl** | ***g*** | **se of *g*** | **Bayes**  **Factor**  **Uniform** | **Bayes**  **Factor**  **Normal** | **Bayes**  **Factor**  **½ Normal** |
| --- | --- | --- | --- | --- | --- | --- | --- | --- | --- | --- | --- |
| 1A: Priming effect, all methods | GLM#1 | .006 | .022 | .396 | 474 | 479 | 0.02 | 0.06 | .12 | .06 | .19 |
| 1B: Implicit priming effect, all subjects | GLM#2 | -.019 | .028 | .754 | 289 | 312 | -0.06 | 0.08 | .07 | .05 | .12 |
| 1C: Explicit priming effect, all subjects | GLM#3 | .031 | .034 | .183 | 185 | 167 | 0.10 | 0.11 | .38 | .25 | .57 |
| 2A: Interaction with religiosity,  all methods | GLM#1 | .008 | .046 | .431 | 474 | 479 | - | - | 1.01** | N/A | N/A |
| 2A: Interaction with religiosity,  all methods | GLM#1 | .008 | .046 | .431 | 474 | 479 | - | - | .26*** | N/A | N/A |
| 2A: Priming effect, all methods,  Religious subs only | GLM#1 | .01 | .026 | .356 | 302 | 320 | 0.03 | 0.08 | .18 | .09 | .28 |
| 2A: Priming effect, all methods,  Non-religious subs only | GLM#1 | .002 | .036 | .478 | 172 | 159 | 0.01 | 0.11 | .15 | .08 | .24 |
| 2B: Interaction with religiosity,  Implicit priming only | GLM#2 | -.044 | .055 | .788 | 289 | 312 | - | - | 0.00† | N/A | N/A |
| 2B: Interaction with religiosity,  Implicit priming only | GLM#2 | -.044 | .055 | .788 | 289 | 312 | - | - | .16*** | N/A | N/A |
| 2B: Implicit priming effect,  Religious subs only | GLM#2 | -.041 | .032 | .898 | 187 | 209 | -0.13 | 0.10 | .07 | .06 | .11 |
| 2B: Implicit priming effect,  Non-religious subs only | GLM#2 | .003 | .045 | .473 | 102 | 103 | 0.01 | 0.14 | .17 | .10 | .27 |
| 2C: Interaction with religiosity,  Explicit priming only | GLM#3 | .06 | .068 | .190 | 185 | 167 | - | - | 1.31** | N/A | N/A |
| 2C: Interaction with religiosity,  Explicit priming only | GLM#3 | .06 | .068 | .190 | 185 | 167 | - | - | .82*** | N/A | N/A |
| 2C: Explicit priming effect,  Religious subs only | GLM#3 | .061 | .041 | .068 | 115 | 111 | 0.20 | 0.13 | 1.08 | 1.03 | 1.48 |
| 2C: Explicit priming effect,  Non-religious subs only | GLM#3 | .001 | .055 | .494 | 70 | 56 | 0.00 | 0.18 | .25 | .18 | .36 |

*Note: *p*-values are one-tailed

**Bayes factor reflects original pre-registered analytic strategy

***Bayes factor reflects revised analytic strategy

†Bayes factor reflects original pre-registered analytic strategy, but the religious priming effect was in the direction counter to theory, rendering computation of the Bayes factor using pre-registered methods problematic.

**Table S6, Experiment 2 Results Summary, No Subjects Excluded: Interactions with View of God as Authoritarian Figure**

| **Prediction** | **Model** | **Mean**  **Diff** | **SE** | ***p**** | **Bayes**  **Factor**  **Uniform** |
| --- | --- | --- | --- | --- | --- |
| “A” Scale moderates priming effect, all methods | GLM#4 | .002 | .023 | .465 | 1.13** |
| “A” Scale moderates priming effect, all methods | GLM#4 | .002 | .023 | .465 | 1.00*** |
| “A” Scale moderates priming effect,  Implicit priming only | GLM#5 | .015 | .027 | .299 | 0.00† |
| “A” Scale moderates priming effect,  Implicit priming only | GLM#5 | .015 | .027 | .299 | 1.47*** |
| “A” Scale moderates priming effect,  Explicit priming only | GLM#6 | -.026 | .040 | .746 | .81** |
| “A” Scale moderates priming effect,  Explicit priming only | GLM#6 | -.026 | .040 | .746 | .57*** |

Note: *p*-values are one-tailed

**Bayes Factor calculated from High/Low split

***Bayes Factor calculated from High/Low split; non-preregistered analytic approach

†Bayes Factor calculated from High/Low split; the religious priming effect was in the direction counter to theory, rendering computation of the Bayes factor using pre-registered methods problematic.

**Table S7. Experiment 1, Results of Nonparametric Tests**

| **Prediction** | **Median**  **Prime** | **Median Control** | **Mann-Whitney *U*** | **Mann-Whitney *z*** | **Mann-Whitney *p*** | **Mann-Whitney *r*** |
| --- | --- | --- | --- | --- | --- | --- |
| Priming effect, all methods & subjects | 0.2 | 0.3 | 64,522.50 | -0.441 | 0.659 | -0.02 |
| Implicit priming effect, all subjects | 0.2 | 0.3 | 28,597.50 | -0.955 | 0.339 | -0.04 |
| Explicit priming effect, all subjects | 0.5 | 0.3 | 7,106 | 0.851 | 0.395 | 0.05 |
| Priming effect, all methods, Religious subs | 0.3 | 0.3 | 27,037 | -0.379 | 0.705 | -0.02 |
| Priming effect, all methods, Non-religious subs | 0 | 0.1 | 7934 | -0.129 | 0.898 | -0.01 |
| Implicit priming effect, Religious subs only | 0.3 | 0.3 | 11792.5 | -0.81 | 0.418 | -0.05 |
| Implicit priming effect, Non-religious subs only | 0 | 0.1 | 3543.5 | -0.621 | 0.535 | -0.05 |
| Explicit priming effect, Religious subs only | 0.5 | 0.35 | 3067.5 | 0.787 | 0.431 | 0.06 |
| Explicit priming effect, Non-religious subs only | 0.1 | 0.05 | 860.5 | 0.717 | 0.473 | 0.08 |

**Table S8. Experiment 1: Results of Nonparametric Tests, No Subjects Excluded**

| **Prediction** | **Mdn1 Prime** | **Mdn2 Ctrl** | **Mann-Whitney U** | **Mann-Whitney z** | **Mann-Whitney p** | **Mann-Whitney r** |
| --- | --- | --- | --- | --- | --- | --- |
| Priming effect, all methods & subjects | 0.2 | 0.3 | 112,509.00 | 0.031 | 0.975 | 0.00 |
| Implicit priming effect, all subjects | 0.2 | 0.3 | 42,480.00 | -0.922 | 0.357 | -0.04 |
| Explicit priming effect, all subjects | 0.45 | 0.3 | 16,655 | 1.217 | 0.224 | 0.06 |
| Priming effect, all methods, Religious subs | 0.3 | 0.3 | 47,990 | -0.058 | 0.954 | 0.00 |
| Priming effect, all methods, Non-religious subs | 0.1 | 0.1 | 13139 | 0.037 | 0.97 | 0.00 |
| Implicit priming effect, Religious subs only | 0.2 | 0.3 | 17829 | -0.71 | 0.478 | -0.04 |
| Implicit priming effect, Non-religious subs only | 0 | 0.1 | 4955 | -0.87 | 0.384 | -0.06 |
| Explicit priming effect, Religious subs only | 0.5 | 0.3 | 7323.5 | 0.856 | 0.392 | 0.06 |
| Explicit priming effect, Non-religious subs only | 0.3 | 0 | 1920 | 1.091 | 0.275 | 0.10 |

**Table S9. Experiment 2: Results of Nonparametric Tests**

| **Prediction** | **Mdn1 Prime** | **Mdn2 Ctrl** | **Mann-Whitney *U*** | **Mann-Whitney *z*** | **Mann-Whitney *p* (2-tailed)** | **Mann-Whitney *r*** |
| --- | --- | --- | --- | --- | --- | --- |
| Priming effect, all methods & subjects | 0 | 0 | 70091.50 | -0.772 | 0.440 | -0.03 |
| Implicit priming effect, all subjects | 0 | 0 | 31054.50 | -1.175 | 0.240 | -0.05 |
| Explicit priming effect, all subjects | 0.1 | 0 | 7873.00 | 0.455 | 0.649 | 0.03 |
| Priming effect, all methods, Religious subs | 0 | 0 | 29595.50 | -0.351 | 0.726 | -0.02 |
| Priming effect, all methods, Non-religious subs | 0 | 0 | 8316.00 | -0.659 | 0.510 | -0.04 |
| Implicit priming effect, Religious subs only | 0 | 0.05 | 12813.00 | -1.312 | 0.189 | -0.07 |
| Implicit priming effect, Non-religious subs only | 0 | 0 | 3728.00 | -0.138 | 0.891 | -0.01 |
| Explicit priming effect, Religious subs only | 0.1 | 0 | 3482.00 | 1.47 | 0.142 | 0.12 |
| Explicit priming effect, Non-religious subs only | 0 | 0 | 899.00 | -0.947 | 0.344 | -0.10 |

**Table S10. Experiment 2: Results of Nonparametric Tests, No Subjects Excluded**

| **Prediction** | **Mdn1 Prime** | **Mdn2 Ctrl** | **Mann-Whitney *U*** | **Mann-Whitney *z*** | **Mann-Whitney *p*** | **Mann-Whitney *r*** |
| --- | --- | --- | --- | --- | --- | --- |
| Priming effect, all methods & subjects | 0 | 0 | 114504.00 | -0.163 | 0.87 | -0.01 |
| Implicit priming effect, all subjects | 0 | 0 | 43949.50 | -1.027 | 0.305 | -0.04 |
| Explicit priming effect, all subjects | 0.05 | 0 | 16341.00 | 0.961 | 0.336 | 0.05 |
| Priming effect, all methods, Religious subs | 0.05 | 0.025 | 48044.00 | -0.126 | 0.899 | -0.01 |
| Priming effect, all methods, Non-religious subs | 0 | 0 | 13673.00 | -0.001 | 0.999 | 0.00 |
| Implicit priming effect, Religious subs only | 0 | 0 | 11118.00 | -1.284 | 0.199 | -0.06 |
| Implicit priming effect, Non-religious subs only | 0 | 0 | 5252.00 | -0.002 | 0.998 | 0.00 |
| Explicit priming effect, Religious subs only | 0.1 | 0 | 7103.50 | 1.509 | 0.131 | 0.10 |
| Explicit priming effect, Non-religious subs only | 0 | 0 | 1924.50 | -0.179 | 0.858 | -0.02 |
